# Composition-driven archetype dynamics in polyoxovanadates†

**DOI:** 10.1039/d2sc01004f

**Published:** 2022-04-29

**Authors:** Aleksandar Kondinski, Maren Rasmussen, Sebastian Mangelsen, Nicole Pienack, Viktor Simjanoski, Christian Näther, Daniel L. Stares, Christoph A. Schalley, Wolfgang Bensch

**Affiliations:** Department of Chemical Engineering and Biotechnology, University of Cambridge Philippa Fawcett Drive S CB3 0AS UK aleksandar@kondinski.com; Institut für Anorganische Chemie, Christian-Albrechts-Universität zu Kiel 24118 Kiel Germany wbensch@ac.uni-kiel.de; Primer affiliate of University of Chicago Master Program Chicago IL USA; Institut für Chemie und Biochemie der Freien Universität Berlin Arnimallee 20 14195 Berlin Germany c.schalley@fu-berlin.de

## Abstract

Molecular metal oxides often adopt common structural frameworks (*i.e.* archetypes), many of them boasting impressive structural robustness and stability. However, the ability to adapt and to undergo transformations between different structural archetypes is a desirable material design feature offering applicability in different environments. Using systems thinking approach that integrates synthetic, analytical and computational techniques, we explore the transformations governing the chemistry of polyoxovanadates (POVs) constructed of arsenate and vanadate building units. The water-soluble salt of the low nuclearity polyanion [V_6_As_8_O_26_]^4−^ can be effectively used for the synthesis of the larger spherical (*i.e.* kegginoidal) mixed-valent [V_12_As_8_O_40_]^4−^ precipitate, while the novel [V_10_As_12_O_40_]^8−^ POVs having tubular cyclic structures are another, well soluble product. Surprisingly, in contrast to the common observation that high-nuclearity polyoxometalate (POM) clusters are fragmented to form smaller moieties in solution, the low nuclearity [V_6_As_8_O_26_]^4−^ anion is *in situ* transformed into the higher nuclearity cluster anions. The obtained products support a conceptually new model that is outlined in this article and that describes a continuous evolution between spherical and cyclic POV assemblies. This new model represents a milestone on the way to rational and designable POV self-assemblies.

## Abstract 2

Systems-based elucidation of the polyoxovanadate speciation reveals that heterogroup substitution can transform spherical kegginoids into tubular architectures in a programmable manner.

## Graphical abstract

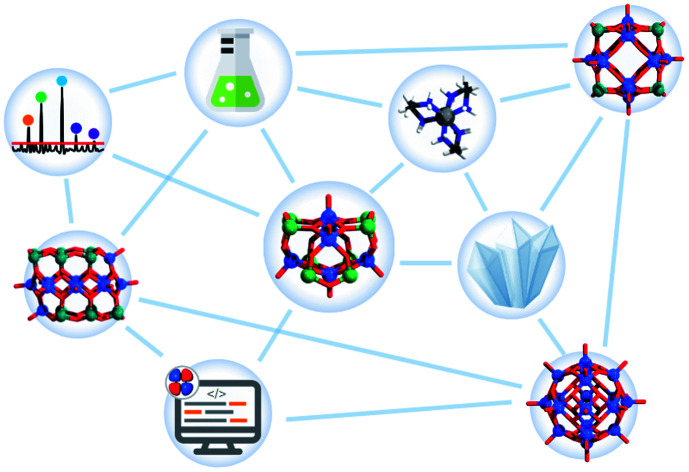

## 1 Introduction

Polyoxometalates (POMs) are forms of molecular metal oxides conventionally built of early transition metals (V, Mo, W, Nb and Ta) in high oxidation states.^1,2^ In terms of bonding, the construction of POMs exclusively relies on metal–oxygen bonds,^3^ which may differ in terms of inertness.^4^ The metal and oxygen atoms often assemble into a small number of skeletal frameworks resulting in representative structural archetypes.^5^ Keggin,^6,7^ Wells-Dawson,^8^ Anderson-Evans^9^ and Lindqvist^10^ POMs are forms of intensely investigated POM archetypes instantiated by thousands of POM formulations and have a profound technological impact in catalysis,^11,12^ life-science^13,14^ and variety of energy storage^15–17^ and energy conversion “POMtronics”.^18–20^

**Fig. 1:**
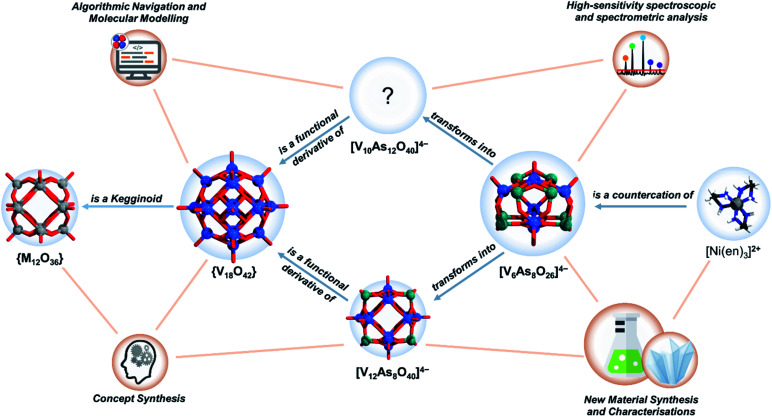
Schematic representation the system of relationally interconnected inorganics (blue circles) and the network of techniques (brown circles) used for their study and description. For clarity the most important relations projected. Colour code: V = blue, M/Ni = grey, O = red, As = green, C = black, N = dark blue, H = white.

As POMs constitute the most complex inorganic materials,^21^ a significant portion of their development relied on probabilistic synthetic self-assembly approaches.^22^ Therefore, a significant challenge in the history of POM chemistry was to decipher the relationship between archetypes and how they are interconnected.^23^ A focal point of these studies has been the description and instantiation of the Keggin structure,^24^ which in its most generic and idealised form {M_12_O_24_} can be described as a virtual rhombicuboctahedral (rbc) graph made of twenty-four bridging μ_2_-O ligands connecting a cuboctahedral network of twelve metal centres (Fig. 1).^6^ Among Mo- and W-based POMs, the framework can incorporate a central {XO_4_} hetero-group where X = P, S, Si, Ge, Al, As *etc.*, while each metal addendum centre is also oxo-terminated, leading to the classical {(XO_4_)M_12_O_36_} Keggin species.^6^ The Keggin-type structures can undergo metal addenda substitution with other heteroelements or hetero-groups, giving rise to different configurational polyanionic compositions.^25–27^ Depending on the nature and dimension of the substituting units, the energetic differences between different configurations can be minimised or maximised, which ultimately affects the isomerisation and the speciation of the formed and isolated POMs.^26^

The kegginoidal {M_12_O_24_} can undergo further saturation with the formal addition of six metal centres.^7^ The metal centres occupy the vacant sites described by a square of oxo-ligands in the rhombicuboctahedral framework forming the {M_18_O_24_} topology with *O*_h_ symmetry. The latter topology is designated with “α”, as a rotation of one pentametalate cupola transforms the structure into the pseudo-rhombicuboctahedral shape, or the *D*_4d_ symmetric β-{M_18_O_24_} isomer.^7^ In its native form, α-{M_18_O_24_} has been instantiated for late transition metal-based POMs (M = Cu^2+^, Ni^2+^ and Pd^2+^).^6^ However, both the α- and β-{M_18_O_24_} archetypes are most commonly discovered among polyoxovanadates (POVs), where addenda metals are actually vanadyl cations (*i.e.* M = [VO]^*n*+^), that is [V_18_O_42_]^12−^ polyanions.^28,29^

In comparison to the Mo–O and W–O bonds in POMs, the V–O bonds in POVs are less inert.^2,30^ This difference is often attributed to the higher charge density (*ρ*_CD_) accumulated by POVs that derives from the smaller atomic radius and lower highest oxidation number of V cations. An illustrative example is the octahedral hexavanadates {M_6_O_19_} in which for M = V^V^*ρ*_CD_ = 31.738C mol dm^−3^ while for M = Mo *ρ*_CD_ = 8.403C mol dm^−3^.^31,32^ The increased lability has been a challenge as it normally requires the presence of multiple vanadyl cations within the forming species to ensure their stability (*e.g.* as in [V_18_O_42_]^12−^). However, at the same time, the lability provides an opportunity for the exchange of oxo by alkoxo ligands which eventually has led to a plethora of organic functionalization and sustainable electron storage solutions.^33,34^ The higher lability makes POVs also more transformable, exemplified by the controlled isomeric transformation between spherical, hemispherical and tubular [V_12_O_32_]^4−^ POVs.^35,36^

Our ultimate research interest in POVs is to understand the principles that govern their formation and to apply them towards rational design and electronic structure engineering. In this endeavour over the past years, we have successfully implemented new functionalities to the POVs by changes in the composition of the heterogroup,^37^ organo-functionalisation,^38^ and the preparation of water-soluble hetero-POV salts enabling further aqueous studies with electrospray ionisation mass spectrometry (ESI-MS).^39–41^ In this work, we focus on the substitution pattern and the archetype transformations, when the POV system becomes saturated with arsenate building units. Using solvothermal synthesis, we develop a pathway to a water-soluble, As-rich POV [V_6_As_8_O_26_]^4−^ exhibiting an ideal *D*_2d_ point group symmetry. Over two decades ago, Hervé and co-workers reported the formation of [V_6_As_8_O_26_]^4−^,^42^ but this As-POV did not receive much attention from the community. Following our experience in the area, we originally hypothesised that solvothermal synthesis and use of bulkier water-soluble cations can enable high yields and better solubility of [V_6_As_8_O_26_]^4−^ salts. Consequently, we have successfully prepared [Ni(en)_3_]_2_[V_6_As_8_O_26_] (1) (V_6_As_8_) where en = ethylenediamine, with high water solubility (*ca.* 20 g L^−1^) in high yields and herein we describe the process of its preparation and characterization. Fresh aqueous solutions of V_6_As_8_ are suitable for ESI-MS studies and they unsurprisingly show the *m*/*z* signal corresponding to [V_6_As_8_O_26_]^4−^. However, aging of the V_6_As_8_ solutions show depletion of the ESI-MS signal of [V_6_As_8_O_26_]^4−^, which usually would be associated with decomposition of the primary POM into smaller building units. However, further studies of the aging V_6_As_8_ solution led to the speciation of two other As-POVs, which based on their composition can be described as substituted, but unsaturated species. First, concentrated solutions of V_6_As_8_ led to deposition of a crystalline solid containing kegginoidal [V_12_As_8_O_40_(H_2_O)]^4−^ (2) species (V_12_As_8_). Second, time-dependent ESI-MS measurements of aging V_6_As_8_ solutions showed that a new signal corresponding to an arsenate-rich [V_10_As_12_O_40_]^4−^ (3) species emerges with the gradual depletion of the signal corresponding to [V_6_As_8_O_26_]^4−^.

To resolve the structure of [V_10_As_12_O_40_]^4−^ using computational brute force would require the simulation of thousands of different POV scenarios that surpasses any current achievement in the field.^43,44^ However, empirical intelligence and systems thinking combined with care for retention of local coordination environments (*i.e.* concept synthesis) can provide a significantly constrained direction in which suitable models can be discovered (see Fig. 1). Following this, we have derived a model for [V_10_As_12_O_40_]^4−^, which shows promise of being a viable structural proposal. The proposed structure features structural aspects of both the kegginoidal and the cyclic (*i.e.* tubular) archetype, providing a broader understanding on the structural principles governing the assembly of POVs.

## 2 Methods

### 2.1 Synthesis and characterisation

The optimal conditions for the synthesis of [Ni(en)_3_]_4_[V_6_As_8_O_26_] were systematically developed. All syntheses were performed in Duran glass tubes with a volume of 11 mL. First, we used a mixture of 156.9 mg (1.34 mmol) NH_4_VO_3_, 209.7 mg (1.06 mmol) As_2_O_3_, 157.0 mg (0.66 mmol) NiCl_2_·6H_2_O, 1.7 mL ethylenediamine (en) and 2.3 mL H_2_O which was heated to 150 °C for 3 d. After recovering the solid product by filtration, green rod-like crystals and small brown aggregates were obtained in a 2 : 1 ratio. The green crystals could be manually separated from the aggregates and a yield of *ca.* 44% based on NH_4_VO_3_ was estimated. To increase the yield, the amount of As_2_O_3_ was increased to 1.19 mmol (236.0 mg) keeping the amounts of the other ingredients constant, and after heating at 150 °C for 3 d, the yield of the green crystals increased to 86% based on NH_4_VO_3_. An upscaling was done using 627.1 mg (5.36 mmol) NH_4_VO_3_, 944.2 mg (4.77 mmol) As_2_O_3_, 627.5 mg (2.64 mmol) NiCl_2_·6H_2_O in 6.8 mL en and 9.2 mL H_2_O affording 1282.6 mg of the title compound after heating the slurry in a Teflon-lined steel autoclave (inner volume: 30 mL) at 150 °C for 4 d (yield based on NH_4_VO_3_: 78%).

Storing a saturated aqueous solution of [Ni(en)_3_]_4_[V_6_As_8_O_26_] in a glass tube closed with a perforated cap for more than 10 d at room temperature afforded precipitation of a blue crystalline powder. The EDX analysis yielded an atomic ratio V : As : Ni of 6 : 3 : 1. Further characterisations using X-ray powder diffraction, energy dispersive X-ray spectroscopy, elemental analysis, UV-Vis, FT-IR, and stability experiments were performed (see ESI† for details).

### 2.2 Crystal structure determination

#### 2.2.1 [Ni(en)_3_]_2_[V_6_As_8_O_26_] (1)

Data collection was performed with an imaging plate diffraction system (IPDS-2) from STOE & CIE using Mo K_α_-radiation. Structure solution was performed with SHELXS-97,^45^ and structure refinement was performed against *F*^2^ using SHELXL-2018.^46^ A numerical absorption correction was applied using the X-RED and X-SHAPE programs of the X-AREA program package. All non-hydrogen atoms were refined with anisotropic displacement parameters. All C–H hydrogen atoms as well as the N–H hydrogen atoms were positioned with idealized geometry and were refined isotropically with *U*_iso_(H) = 1.2 *U*_eq_(C/N) using a riding model. Selected crystal data and refinement results are listed in Table S1.† Fig. S6† shows the structure of the anion and cation drawn at the 50% probability level together with the atom labelling.

#### 2.2.2 [Ni(en)_3_]_2_[V_12_As_8_O_40_(H_2_O)]·4H_2_O (2)

X-ray powder diffraction data for *ab initio* structure solution were collected at the X04SA-MS beamline of the Swiss Light Source (SLS, Paul Scherrer Institute, Villigen).^47^ The sample was loaded in a 0.3 mm glass capillary (Hilgenberg, Spezialglas Nr. 14) and measured in Debye–Scherrer geometry with a wavelength of 0.5639132 Å. The exact wavelength was refined against a measurement of Si powder. The instrumental line broadening was determined on a PXRD pattern of NAC powder (Na_2_Al_2_Ca_3_F_14_) *via* a LeBail fit using a Thompson-Cox-Hastings pseudo-Voigt profile. Indexing,^48^ charge flipping, simulated annealing,^49^ and Rietveld refinements were carried out with TOPAS Academic V 6.0.^50^ The background was modelled using an artificial phase with strongly broadened lines to account for the broad humps, while sample reflection broadening was best described as anisotropic strain broadening. Full details of the structure solution strategy and of the Rietveld refinements including a plot showing the arrangement of the cluster anions and a table summarizing the refinement are given in the ESI (Table S2 and Fig. S7†).

### 2.3 Electrospray mass-spectrometry

ESI-MS was performed with a Water's Synapt G2-S ion mobility Q-TOF mass spectrometer (Manchester, UK) equipped with a Z-spray electrospray ionisation source. Measurements were done in the negative mode with a capillary voltage of 2.08 kV with sample cone and source offset set to 38 and 45 V, respectively. A source temperature of 100 °C was used while the desolvation gas temperature was set to 250 °C. Exact masses were measured using leucine enkephalin as lock mass introduced as an internal standard *via* a separate ion source every 10 seconds. Samples were prepared with concentrations of 100 μM and injected with a flow rate of 5 μL min^−1^. Samples were prepared with either H_2_O, D_2_O (99.90% D, Eurisotop), or H_2_^18^O (97% ^18^O, Cambridge Isotope Laboratories). Samples were stored at 4 °C and measured after different reaction time intervals as indicated in the spectra.

### 2.4 Computational modelling

Density functional theory (DFT) calculations were carried out with the Amsterdam Density Functional program (AMS 2021).^51,52^ Numerical integration was performed using Becke grid integration.^53,54^ Geometry optimization was carried using GGA Becke exchange^55^ and the Perdew 86 correlation^56^ (BP) functional and all-electron Slater basis sets of triple-ζ quality with one polarization function (TZP).^57^ The spin-unrestricted (= *U*) formalism was used for all open-shell electronic systems. Scalar relativistic effects were accounted for using the zeroth-order regular approximation (ZORA).^58–60^ Solvation effects were introduced using the Conductor-like Screening Model (COSMO) with the default parameters for water with correction for the outlying charge.^61,62^ After the geometry optimization at the COSMO/ZORA-Scalar-UBP86/TZP level, we carried unrestricted single-point calculations using the hybrid B3LYP functional,^63^ which describes the electronic structure as in hetero-POVs yielding reliable atomic spin populations at the metal sites.^64,65^

### 2.5 Enumeration and generation of substitutive configurations

The different substitutions corresponding to configomeric substitutions in α-/β-{V_18−*x*_(As_2_O)_*x*_O_42_} are modelled algorithmically (Table 1), as a “two-colour” graph colouring problem.^66,67^ In the graph colouring problem the first colour corresponds to a substituted site, while the second colour to an unsubstituted site in 3D rhombocuboctahedral (rbc) and pseudo-rhombocuboctahedral (prbc) graphs derived from the α-/β-{V_18_O_42_} structures (see Fig. S13†). In these models, the generation and enumeration of the different two-colour configurations for rbc- and prbc-graphs are under the action of the spatial orthogonal group in three dimensions (*i.e.* SO(3)).^68^ The distinct configurations each represent an orbit, *i.e.* a subset of different colourings that are equivalent up to rotation. The spatial orthogonal group in three dimensions (*i.e.* SO(3)) is of the form *r*_1_^i1^*r_2_*^i2^*…*r_*n*_^in^, where *r*_1_, *r*_2_,…*r*_*n*_ represent the group generators. The group generators for SO(3) of the rbc and prbc-graphs are manually encoded, and then the program automatically constructs all possible distinct rotations out of them. In this way, the SO(3) group is formed as all possible products of its generators up to the powers of their respective orders. The generated combinatorial elements are kept in a set, in order to ensure that degeneracies are counted only once. Once the group of rotations is obtained, the orbits under its action are enumerated. In order to execute the isomer enumeration, we begin with an empty set S corresponding to all possible classes (*i.e.* orbits). Then one iterates over all possible colourings of the object with a fixed number of substituting sites. For each “substitution” configuration, one iterates over SO(3), one element at a time, say g, and then calculates *g*·*c*. Thus at the end of the iteration through SO(3), it is ensured that a unique and unaccounted-for colouring *c* has been found and is added to S. This is implemented in the Python 3 programming language and the program is accessible *via* the git repository dedicated to this project (https://www.github.com/digital-chemistry/pov_isomeriser).

**Table 1:** Algorithm of the “pov-isomeriser” referring to “black” colourings as substituted and “white” colourings of unsubstituted sites in α-/β-{V_18−*x*_(As_2_O)_*x*_O_42_} (*i.e.* rbc and prbc graphs respectively)

Input: Hard-code the rotation generators as Python dictionaries		
Output: Sets of configurations corresponding to all possible substitution scenarios in α-/β-{V_18−*x*_(As_2_O)_*x*_O_42_}		
1	**begin**	construct all distinct rotations out of the generators by chaining the dictionaries
2		S ← {} initialize the empty set of different orbits
3		for *b* = 1: V/2 (iterating over possible values for the black vertices)
4		for each subset *Σ* of *b* vertices out of all V vertices
5		take a fresh, ground state object O with all white vertices
6		colour the vertices of O which are in *Σ* black, thus getting the configuration *g*
7		for each rotation r in SO(3), including the identity
8		apply *r* to *g*, if *rg* is not in S, add it to S
9		**end**
10	**return S**	

## 3 Results and discussion

### 3.1 Structural considerations – concept synthesis

Substitution of {VO} with p-block hetero-groups in [V_18_O_42_]^12−^ has been a major way to derive different numbers of V atoms and has paved the way to engineering magnetic POVs.^69,70^ In principle, all eighteen V centres in [V_18_O_42_]^12−^ can be formally substituted, however empirical studies show that this is not the case. Namely, the six vanadyl [VO]^2+^ cations (here for didactical clarity labelled alphabetically [*a*–*f*] and not associated with the kegginoidal framework) act solely as saturating or stabilising units. However, these units can be formally removed in both α-/β-[V_18_O_42_]^12−^ leading to unsaturated species such as {V_16_O_40_}^71^ and {V_14_O_38_}.^72^ Theoretically, the sites of unsaturation create destabilisation which motivate further configurational and archetypal rearrangements.^73^ “Decoding” the interdependence of these rearrangements is currently very challenging and we are not aware of any broader systematic study. However, rearrangements may be enforced by external factors that enhance stability, crystal packing and conformational adaptability to supramolecular interactions. In this context, the highly unsaturated {V_14_O_38_} kegginoid (Fig. S1d†) is known to be able to incorporate a monoatomic Cl^−^,^72^ however while its tubular confirmer {V_14_O_38_} (Fig. S1e†),^73^ appears as conformationally more robust structure able to accommodate three and four atomic anions (*e.g.* N_3_^−^, OCN^−^ and NO_3_^−^) with higher thermochemical radii.^74^

**Fig. 2:**
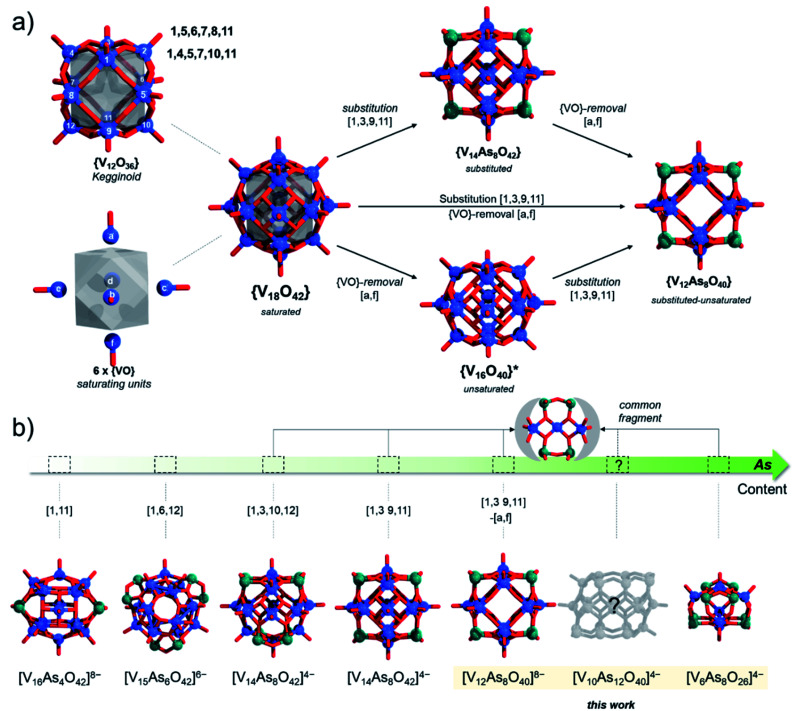
Combination of ball-and-stick and schematic illustrations presenting (a) from left to right: the enumeration the enumeration of the eighteen V centres in the kegginoidal α-{V_18_O_42_}, the kegginoidal α-{V_18_O_42_} archetype, its substituted, unsaturated and substituted-unsaturated derivatives. For {V_16_O_40_}, the experimentally known structure derives from β-{V_18_O_42_}; (b) gradual ordering of the different As-POVs according to increasing arsenic content. The substitution and {VO} removal configurations from {V_18_O_42_} are provided in parentheses. Colour code: V = blue, O = red and As = green spheres.

In α-[V_18_O_42_]^12−^, a formal exchange occurs at the twelve metal sites belonging to the core kegginoidal framework. For clarity, we enumerate these sites as [1−12] (Fig. 2). In a formal exchange, a {VO} unit is substituted by a bent hetero group such as an {As_2_O} group. As one can anticipate, this will lead to a series of configurationally diverse {V_18−*x*_(As_2_O)_*x*_O_42−*x*_} systems, which can be further more compactly written as {V_18−*x*_(As_2_)_*x*_O_42_}. The combinatorial configurational problem associated with {V_18−*x*_(As_2_)_*x*_O_42_} becomes even more complicated, when the two parameters of saturation and substitution are combined (Fig. 2). An example is the mixed-valent [V_12_As_8_O_40_]^4−^ polyanion, which is formally derived by substitution of four {VO} with four {As_2_O} groups followed by two subsequent {VO} unit removals.^75^ However, despite these combinatorial challenges, only a handful of structures have been isolated experimentally. One reason may be the steep energy differential between isomeric configurations.^25,76^ Within the set of possible {V_18−*x*_(As_2_)_*x*_O_42_} isomers, structures have been reported for *x* = 2, 3 and 4. When restricting the substitution only over the twelve vanadium sites, for *x* = 2, there can be four substituting possibilities. However, substitution of {VO} with {As_2_O} occurs exclusively on sites [1,11] leading to [V_16_As_4_O_42_]^8−^ with *D*_2h_ symmetry. For *x* = 3, the substitution occurs on the [1,6,12] sites generating a [V_15_As_6_O_42_]^6−^ cluster with *D*_3d_ symmetry. For *x* = 4, substitutions on [1,3,10,12] and [1,3,9,11] are the typical outcomes generating two different [V_14_As_8_O_42_]^4−^ anions with *D*_2d_ and *D*_4h_ symmetry point groups, respectively.^76^ To the best of our knowledge, higher substitutions for As-POVs are not known; however, substitution of six {VO} units (*i.e. x* = 6) is known to occur for borate units at sites [1,4,5,7,10,11] and [1,5,6,7,8,11].^77,78^ If such derivatives existed for As-POVs, those derivatives would be neutral [V_12_As_12_O_42_] species with *D*_3d_ and *D*_2_ point group symmetries respectively (see Fig. S1a and b†). Other substitutions for *x* connecting to the kegginoidal archetype are not known to us; however, a number of boron-rich POVs have been reported to adopt cyclic archetypes where belts of six, ten or twelve V centres are sandwiched between borate heterogroups.^79,80^

### 3.2 Crystal structures and solubility behaviour

**Fig. 3:**
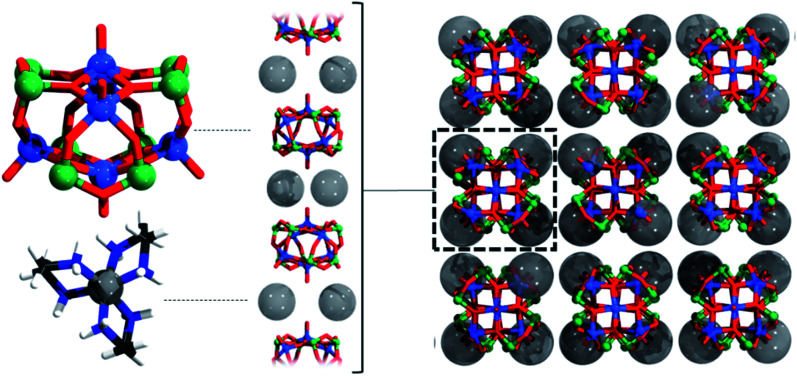
Ball and stick representation of an individual [V_6_As_8_O_26_]^4−^ polyanion and the [Ni(en)_3_]^2+^ dications (left), followed by their hierarchical packing from one (middle) to three dimensions (right). Colour code: V = blue, O = red, As = green, N = dark blue, Ni = grey, C = black, H = white spheres. For clarity, [Ni(en)_3_]^2+^ in the middle and right figure are represented as transparent grey spheres.

The compound, [Ni(en)_3_]_2_[V_6_As_8_O_26_] (1), crystallizes in the tetragonal space group *I*4_1_/*acd*. One V, the Ni and one O atom are located on special positions, while all other unique atoms (one V, two As, six O and all C, H, N atoms) occupy general positions. The two independent V centres are in a fivefold environment of oxygen atoms to form a rectangular VO_5_ pyramid. Three VO_5_ polyhedra share common edges generating trimeric units which are rotated by *ca.* 90° against each other (Fig. 3). Four As_2_O_5_ handle-like bridges composed of corner-sharing AsO_3_ units join the two trimeric moieties yielding the final structure of the cluster shell (Fig. 3), which has a diameter of 8.7 Å. The cavity of the cluster shell is too small to host a guest molecule as observed for larger cluster anions. The V=O bonds to the terminal O atoms are between 1.599(4) and 1.602(5) Å, while those to O atoms in the rectangular plane are longer at 1.916(3) – 2.001(3) Å (Table S3†). The ∠(O–V–O) angles, ranging from 74.92(14)° to 145.88(14)°, indicate a pronounced distortion of the VO_5_ polyhedra (Table S3†). The bond valence sums (BVS) for the two V centres are 4.12 and 4.13, in accordance with the assignment of an oxidation state of +4. The volume of the VO_5_ rectangular pyramids were calculated using the minimum bound ellipsoid (MBE) approach yielding 26.92 (V1) and 26.89 Å^3^ (V2). The shape parameter *S* = −0.11 for the V1-centred polyhedron indicates a slight elongation, while *S* = 0.049 for the V2-centred pyramid suggests a slight compression.^81^ The V⋯V separations in the trimeric units are 3.12 Å, which are at the upper end of data reported for other polyoxovanadate clusters for which V⋯V separations between 2.8 and 3.1 Å were reported.^82–91^ The As–O bonds are between 1.709(3) and 1.814(3) Å with ∠(O–As–O) angles ranging from 95.36(14)° to 102.79(16)° (Table S2†) and ∠(As–O–As) angles of 126.92(19)°. In most As-POVs the As–O bond length are in a relatively narrow range from ≈1.75 to 1.80 Å with an As–O–As angle of 132° to 136°.^92,93^ These analyses of the geometric parameters indicate that the As–O–As angle is slightly strained in the structure of the title compound. We note that the As–O bonds in [*n*Bu_4_]_4_[V_6_As_8_O_26_] (space group: *P*4̄*n*2) scatter between 1.69(1) and 1.81(1) Å with an As–O–As angle of 126.5°, while all other geometric parameters are similar to those observed for the title compound.^42^

The unique Ni^2+^ cation is surrounded by six N atoms of three en ligands in a distorted octahedral environment adopting the Λ-δδδ configuration. The Ni–N bond lengths between 2.115(4) and 2.127(4) Å are in the normal range (Table S4†). The MBE approach resulted in a volume of 39.9 Å^3^ for the NiN_6_ octahedron and in a shape parameter of −0.063 indicating a slight elongation.^94^

The cations and anions are packed along [001] in a layer-like fashion (Fig. 2) and intermolecular N–H⋯O hydrogen bonding interactions are observed (Table S5†). The arrangement of the cations and anions leads to well-shielded cluster anions preventing any weak intermolecular As⋯O interaction.

**Fig. 4:**
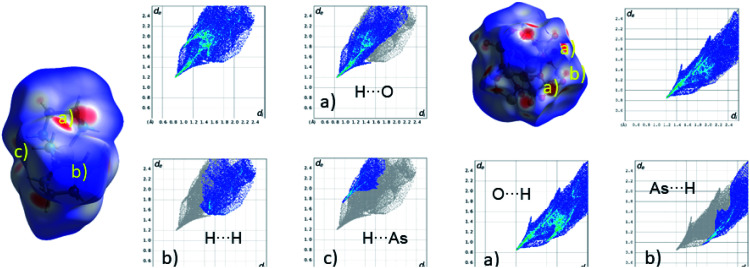
Hirshfeld surface and fingerprint plots of [Ni(en)_3_]^2+^ dications (left) and the polyanionic unit [V_6_As_8_O_26_]^4−^ (right) in the structure of 1. The intermolecular interactions are indicated as red areas on the Hirshfeld surfaces and the distinct interactions are shown in the four fingerprint plots right next to the species. The letters on the Hirshfeld surface (a, b and c) refer to the according letter in the fingerprint plot.

A Hirshfeld surface analysis was performed using Crystal Explorer 17 for gaining more detailed insight into intermolecular interactions.^95–100^ In this approach, a Hirshfeld surface is mapped over *d*_norm_ which is defined as:
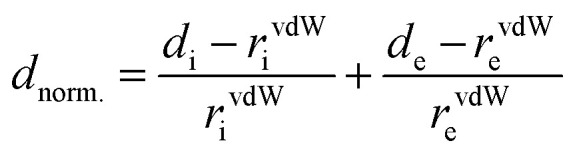
*d*_i_ is the distance of a point on the surface to the nearest nucleus inside the surface; *d*_e_ is the distance from such a point to the next neighbour outside the surface (vdW = van der Waals radius). The red areas on the Hirshfeld surface indicate interatomic distances which are shorter than the sum of the van der Waals radii, whereas white-to blue-coloured areas show interatomic distances longer than the sum of the van der Waals radii. From the 3D Hirshfeld surface, a fingerprint plot can be constructed, which represents a 2D view of the 3D Hirshfeld surface (Fig. 4). For the cation, the main contributions are H⋯H (29.2%), H⋯O (55.6%) and H⋯As (15.1%), while the anion exhibits O⋯H (73.4%) and As⋯H (26.5%) interactions. The relatively large number of intermolecular As⋯H interactions is remarkable. The shortest As⋯H contact is 2.765 Å (N–H⋯As angle: 152.9°) which is shorter than the sum of van der Waals radii (H: 1.10 Å; As: 1.85 Å).^101^ A second As⋯H contact is at 2.903 Å (C–H⋯As angle: 158.4°) which is on the borderline of the sum of the van der Waals radii.

**Fig. 5:**
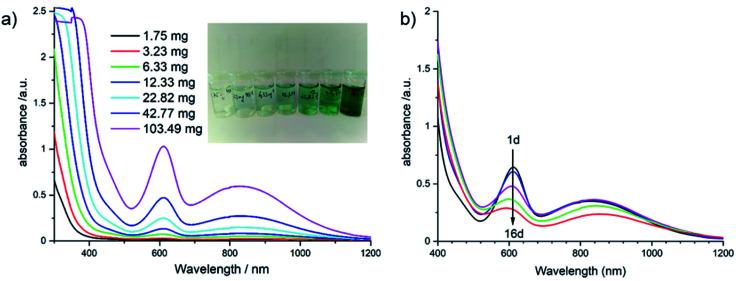
(a) The change of the UV-Vis spectra of aqueous solutions (5 mL) with increasing concentration; (b) the time-dependent evolution of a solution after 1 day until 16 days. The inset in (a) shows the change of the colour with increasing concentration of the solution.

Based on our recent experience with Sb-POVs containing [M(en)_3_]^2+^ cations (M = Ni^2+^, Zn^2+^),^25,39,40,102^ we investigated whether the title compound is soluble in different solvents. The best solubility is observed for water (Fig. 5, left) and the UV-Vis spectrum shows a prominent absorption band at 610 nm and a broad band centred at around 830 nm. For an assignment of this band, one has to consider different contributions from the constituents of the title compound in solution. For example, an aqueous solution of VOSO_4_·5H_2_O containing the VO^2+^ group as model compound shows two bands which are located at 769 and at 625 nm,^103^ and an intense charge transfer (CT) transition is observed at 345 nm. The intact violet [Ni(en)_3_]^2+^ complex displays the most intense band at 545 nm (^3^A_2g_ → ^3^T_1g_(F) transition) and a broad absorption is located at 888 nm (^3^A_2g_ → ^3^T_1g_(F) transition). The third possible electronic transition (^3^A_2g_ → ^3^T_1g_(P)) is in the same energetic region like the CT band of the VO^2+^ ion.^104^ If all en ligands of [Ni(en)_3_]^2+^ are exchanged by H_2_O the hexa–aqua complex results in a green colour and an absorption band at 720 nm. The main band of [Ni(en)_3_]^2+^ shows a continuous shift when en is replaced by H_2_O in [Ni(en)_*x*_(H_2_O)_(6−2*x*)_]^2+^ complexes, and for [Ni(en)(H_2_O)_4_]^2+^ the ^3^A_2g_ → ^3^T_1g_(F) transition occurs at 610 nm.^105^ As can be seen in the inset of Fig. 5 left, the solution turns green with increasing concentration of the title compound suggesting the formation of [Ni(en)(H_2_O)_4_]^2+^. Using the strong absorption at around 610 nm in the UV-Vis spectra the maximum solubility was determined as ≈20 g L^−1^ (see Fig. S4†). The long-term stability was investigated by recording time-dependent UV-Vis spectra (Fig. 5, right). With increasing time, the strong absorption at 610 as well as the less intense and broad band at about 830 nm decrease successively. Using a laser pointer, the Tyndall effect occurs after some time indicating that colloidal particles are formed.

**Fig. 6:**
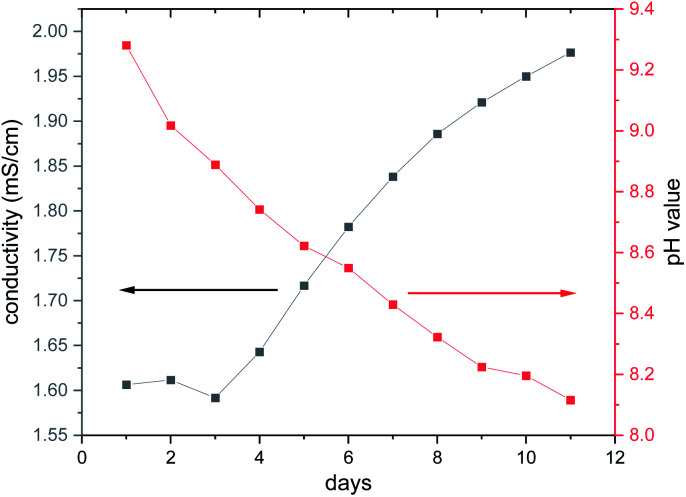
Long-term investigation of the ionic conductivity and the pH value using an aqueous solution (8.26 mmol L^−1^ = 14.9 g L^−1^) of the title compound. The lines are only guide for the eyes.

The long-term behaviour of the dissolved species in the aqueous solution was investigated *in situ* by monitoring the evolution of the ionic conductivity and of the pH value (Fig. 6). At the beginning of the experiment, the conductivity is constant during the first 3 d at around 1.6 mS cm^−1^, while the pH value decreases steadily starting at pH ≈ 9.3. With increasing reaction time, the pH value drops reaching 8.1 after 11 d, whereas the ionic conductivity increases with a value of 1.97 mS cm^−1^ after 11 d. The basic pH value is most probably caused by removal of en ligands and the subsequent reaction of the amine with H_2_O. The second possibility that the basic terminal O^2−^ anions of the cluster anion react with H_2_O cannot be ruled out, as several species were detected in the ESI mass spectra (see below). The precipitation of Ni(OH)_2_ occurs in the pH range from 8.1 to 9.6 to which the pH value drops during the long-term experiment (Fig. 6).

**Fig. 7:**
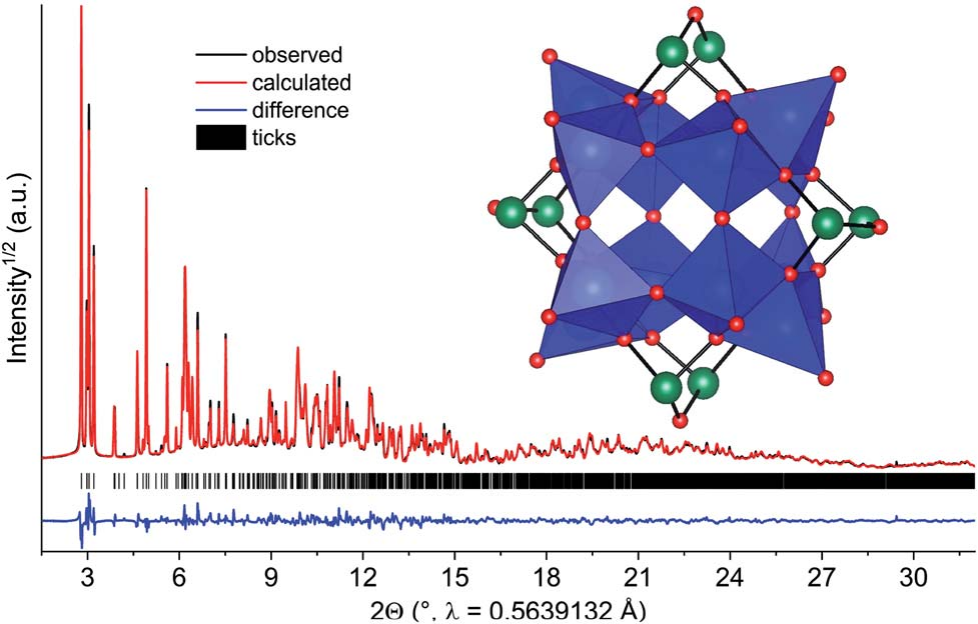
Difference plot of the final Rietveld refinement for [Ni(en)_3_]_2_[As_8_V_12_O_40_(H_2_O)]·4H_2_O with a final *R*_wp_ of 4.7% and *R*_Bragg_ = 3.7%. The inset shows the polyhedral representation of the cluster anion in the structure of 2. Note: the H_2_O molecule in the cavity of the cluster is not displayed. Blue: VO_5_ polyhedra; green: As; red: O.

Compound 2 was obtained as blue polycrystalline powder by stirring an aqueous concentrated solution at *T* = 25 °C 12 d. Since single crystal growth was not successful, the structure was solved and refined from X-ray powder diffraction data. This was particularly challenging since no information on the cluster motif was available. Thus the structure was solved by reciprocal space methods, requiring data of outstanding quality. The difference plot for the final Rietveld-refinement is shown in Fig. 7, demonstrating the excellent agreement between the structure model and experimental diffraction data. The results demonstrate that the mixed-valent compound [Ni(en)_3_]_2_[V_12_As_8_O_40_(H_2_O)]·4H_2_O was formed crystallizing in the monoclinic space group *P*2_1_/*n*.

Six unique V atoms, four independent As atoms and twenty one O atoms are on general positions, while the O atom in the cluster cavity is on a special position. All atoms of the unique Ni^2+^ centred complex are on general positions. The crystal structure of the cluster consists of four trimeric units composed of edge-sharing VO_5_ pyramids, which are joined by common corners (Fig. 7). This connection mode generates niches, which are occupied by As_2_O units and As_2_O_5_ handles are formed. BVS calculation evidence that the V centres are in two different oxidation states and BVS values between 4.16 and 4.55 indicate that the electrons are at least partially delocalized. The presence of V^4+^/V^5+^ is also reflected by the volumes of the VO_5_ pyramids which are between 25.95 and 26.54 Å^3^, *i.e.* clearly smaller than for the polyhedra in 1. We note that mixed-valent [V_12_As_8_O_40_(H_2_O)]^*n*−^ anions are well-known^75,106–108^ and the electronic situations mainly differ in the V^IV^/V^V^ ratios. The geometric parameters like V–O and As–O bond lengths and corresponding angles slightly scatter more than literature data which is most probably due to the lower precision of structural data from XRPD compared to single crystal data. The cluster anions are packed in a body centred fashion (Fig. S7†) and chains directed along [001] are generated by weak intercluster As⋯O interactions (As–O: 3.009 Å). The formation of 2 from 1 can be rationalized comparing the structures of the two clusters: in both structures a secondary building unit with composition V_3_As_2_O_14_ can be identified. This unit is one fourth of the [V_12_As_8_O_40_]^4−^ cluster and interconnection of four such units generates the final anion. The results clearly demonstrate, that a growth in cluster size occurs by ageing an aqueous solution of [Ni(en)_3_]_2_[V_6_As_8_O_26_].

### 3.3 ESI-MS investigations

The behaviour of [Ni(en)_3_]_2_[V_6_As_8_O_26_] in aqueous solution was further investigated by ESI-MS. Initially, the base peak was observed at *m*/*z* 660.45 corresponding to the [V_6_As_8_O_26_]^2−^ ion. This was formed by a loss of the two [Ni(en)_3_]^2+^ counterions and double oxidation during ionisation to form the dianionic species (Fig. S8†). Also present are the singly and non-oxidised species with 1 or 2 protons producing doubly charged ions at *m*/*z* 660.95 and 661.45, respectively. As the elements within the cluster are almost monoisotopic, these assignments were supported *via* measurements of samples in D_2_O showing the formation of the deuterated ions as well as helping identify the charge state (Fig. S9†).

**Fig. 8:**
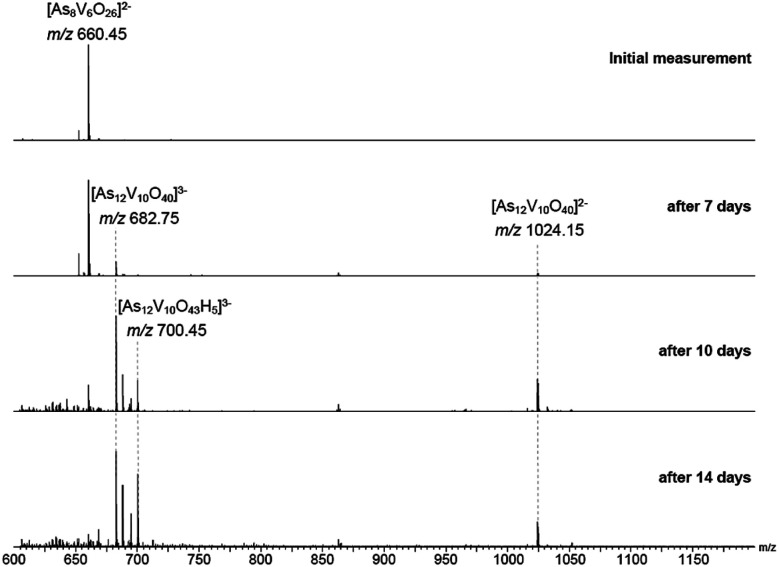
ESI mass spectra of a 100 μM solution of the parent compound in H_2_O recorded after different reaction times. The initial peak at *m*/*z* 660.45 decreases over days with a simultaneous increase of signals at *m*/*z* 682.75 and 1024.15 assigned to the −3 and −2 charge states of {V_10_As_12_O_40_}. Peaks with additional oxygen/water molecules were also observed such as the ion at *m*/*z* 700.45.

Measurements were then repeated at different time intervals over the course of several days to monitor changes in composition over time (Fig. 8). Peaks associated with [V_6_As_8_O_26_]^2−^ slowly disappeared accompanied by the formation of signals for new ions at *m*/*z* 682.75 and 1024.15. These two new ions can be assigned to the −3 and −2 charge states of {V_10_As_12_O_40_} corresponding to a cluster 1.5 times larger than the initial one plus an additional {VO} moiety. Analogous to what was observed for the [V_6_As_8_O_26_]^2−^ ion, the ions at *m*/*z* 682.75 and 1024.15 were also accompanied by different oxidation states with compensating protonation to maintain the charge state (Fig. S10 and S11†). Formation of clusters with additional oxygen atoms and water molecules (such as the ion at *m*/*z* 700.45) were also observed.

**Fig. 9:**
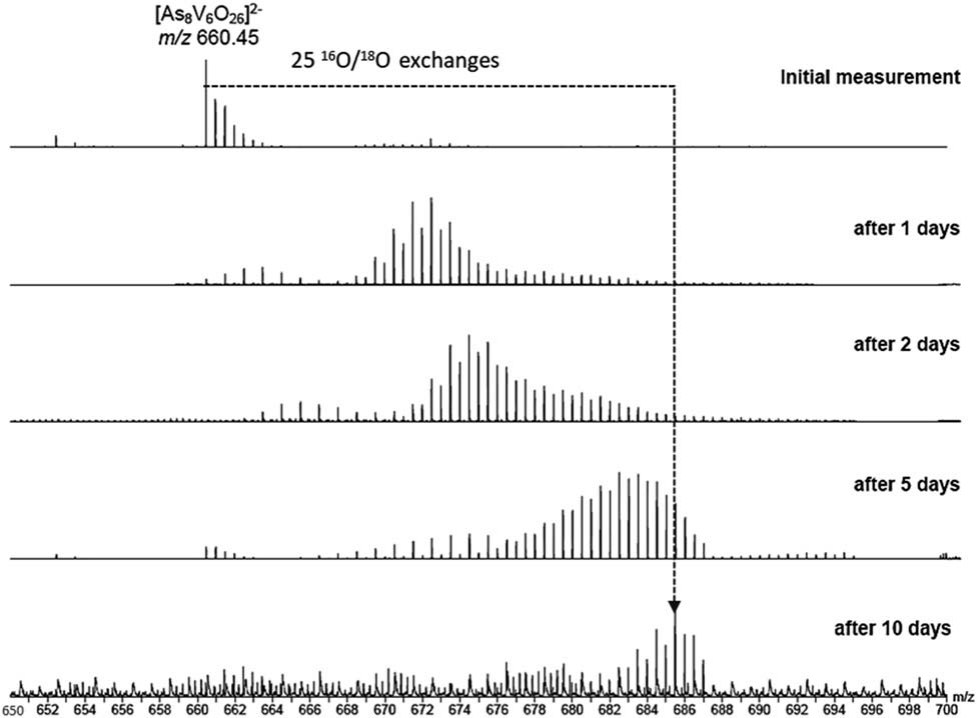
ESI mass spectra of a 100 μM solution of the parent cluster in H_2_^18^O at different reaction times. After about 10 days, a mass shift corresponding to 25 O atom exchanges is achieved.

**Fig. 10:**
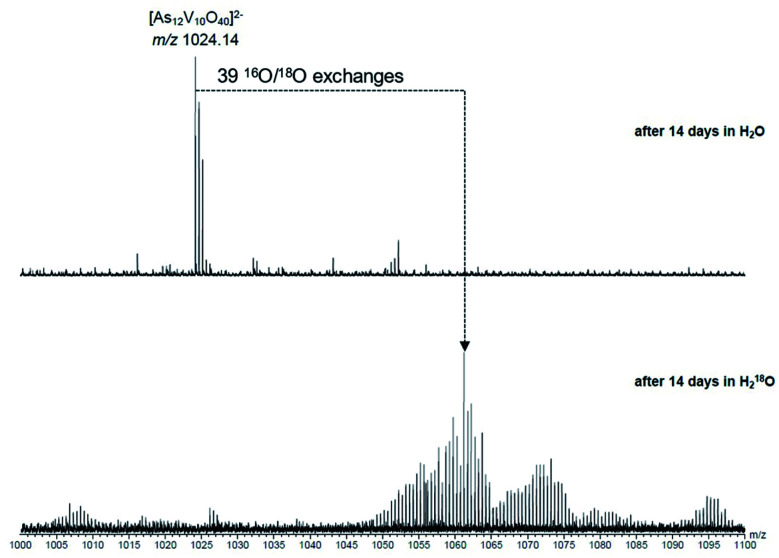
Comparison of samples of the parent cluster in H_2_O (top) and H_2_^18^O (bottom) after 14 days. When corrected for the enrichment of the H_2_^18^O, the number of exchanges is consistent with {V_10_As_12_O_40_}.

To investigate the assignment of {V_10_As_12_O_40_} further, measurements were also performed with samples using H_2_^18^O as the solvent. A cluster in H_2_^18^O will begin to exchange its ^16^O for the ^18^O of the solvent, which can be followed easily by a shift in mass over time. Such measurements can be structure indicative as they can be used to determine the number of exchangeable oxygen atoms present within a cluster.^40^ The [V_6_As_8_O_26_]^2−^ ion at *m*/*z* 660.45 undergoes exchange of 25 oxygen atoms over the course of several days (Fig. 9). As the H_2_^18^O is 97% enriched, this value corrected to 100% enrichment equates to a cluster containing 26 O atoms and thus fits to the assigned ion. As the oxygen exchange is faster than the rate of formation of the new cluster, the {V_10_As_12_O_40_} ions have already undergone a majority of their exchanges by the time of their formation (Fig. S12†). A comparison of the sample in H_2_O and H_2_^18^O after 14 days allows a determination of the number of ^16^O/^18^O exchanges for {V_10_As_12_O_40_} (Fig. 10). The number of exchanges when correcting for the enrichment of the H_2_^18^O, is 40 oxygen atoms, again consistent with the assignment of the new cluster as {V_10_As_12_O_40_}.

Finally, collision induced dissociation (CID) measurements of the different clusters were undertaken to investigate their fragmentation. The fragmentation of {V_6_As_8_O_26_} (Fig. S13†) and {V_10_As_12_O_40_} (Fig. S14†) were similar to one another with both initially losing several {AsO_2_} moieties with subsequent loss of {V_3_O_8_} units.

### 3.4 Algorithm and molecular modelling

**Fig. 11:**
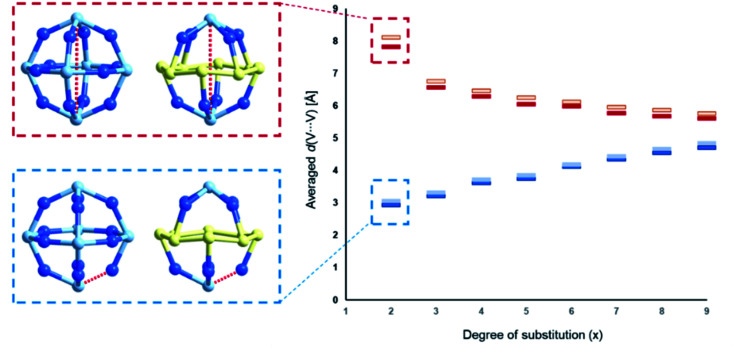
Diagrammatic representation of the maximal (red) and minimal (blue) averaged interatomic (V⋯V) distances within α-{V_18−*x*_(As_2_)_*x*_O_42_} (dark shaded) and β-{V_18−*x*_(As_2_)_*x*_O_42_} (light shaded) systems. Selection of the single interatomic for *x* = 2 for both systems is projected left to the diagram. The structurally inequivalent V centres are coloured differently, namely μ_4_-V = dark blue, μ_3_-V = yellow and μ_2_-V = light blue.

The studies of the isomeric behaviour show that for the α-/β-{V_18−*x*_(As_2_)_*x*_O_42_} system where *x* ranges between 0 and 18, there are in total of 44 512 structural possibilities (see Table 2). Considering that the total number of substitution sites is finite, the number of isomers steadily increases from 0 to 9 and then symmetrically decreases with the increase in *x* (Table 2). For *x* = 0 there is no formal substitution thus we have only one instance of unsubstituted α-/β-{V_18_O_42_}. For *x* = 1, the number of configurationally substituted isomers reflects the number of structurally unequal vanadium sites which in α-{V_18_O_42_} are two and in β-{V_18_O_42_} are three (see Fig. S15†). For *x* = 2 there are 10 configurational possibilities for α-{V_18−*x*_(As_2_)_*x*_O_42_} and 25 for β-{V_18−*x*_(As_2_)_*x*_O_42_}. A large portion of the latter configomeric possibilities are enantiomers. The substitutions for *x* = 2 are also very important as they provide information about the shortest and longest interatomic distances between the substituting sites (*i.e. d*(V⋯V)). In the systems between *x* = 3 and 15 the interatomic distances are expressed as an average of all possible substituted vanadium pairs. When substituting {VO} units with {As_2_O} units, the distance between the newly substituted moieties will be larger than that between the particular centres, however, as it would be computationally very costly to optimise all 44 512 using DFT methods, the averaged distance *d*(V⋯V) within each set of configurations can be representative of an overall trend. Plotting the minimal and maximal *d*(V⋯V) for *x* in the range of 2–9, one observes that as *x* increases, the minimal *d*(V⋯V) rises from *ca.* 3 Å to 5 Å, while the maximal *d*(V⋯V) decreases from *ca.* 8 Å to 6 Å (see Fig. 11). The trend implies that with the increase of the substitution, the bulky {As_2_O} that replace {VO} are enforced on average to come closer to one another which can be destabilising.

**Table 2:** Enumeration of heterogroup substituted isomers of α-{V_18−*x*_(As_2_)_*x*_O_42_} and β-{V_18−*x*_(As_2_)_*x*_O_42_} obtained as a result from the implementation of “pov-isomeriser” algorithm (see Table 1)

M_*x*_M′_18−*x*_	1	2	3	4	5	6	7	8	9
α/rbc	2	10	42	142	380	811	1368	1872	2088
β/prbc	3	25	104	406	1080	2374	3992	5550	6098

Using our algorithm, for *x* = 6 it was calculated that there are 811 α-{V_12_As_12_O_42_} and 2374 topologies deriving from β-{V_12_As_12_O_42_} topologies. The number of topologies will increase even further if we consider a 3-colouring system, one that accounts for substitution and loss of {VO} moieties simultaneously. Duly modelling of those topologies and their optimisation based on DFT would be a “brute force” approach that comes with an enormous computational cost. To tackle this problem, we apply a system approach where suitable configurations emerge when they relationally satisfy a particular set of expectations.

To be aware if a particular *in silico* derived chemical structure methods is experimentally viable structure, Hoffmann and co-workers have outlined a protocol,^109^ which we also apply to evaluate the quality of the derived structural model representative for the [V_10_As_12_O_40_]^4−^ species. For a POM to be observable by ESI-MS, it is expected to exhibit sufficient kinetic stability in terms of bonding energy.^39^ When done in comparison to experimentally confirmed structures, the energy gap between the highest occupied and the lowest unoccupied molecular orbital (*i.e.* HOMO and LUMO, respectively) can be used as an indicator whether the particular structural model is sufficiently plausible to exist or form in nature.

To understand what are the expected ranges for energetic realism in the present context, we consider the set of five fully reduced As-POVs, namely [V_16_As_4_O_42_]^8−^, [V_15_As_6_O_42_]^6−^, α-/β-[V_14_As_8_O_42_]^4−^ and [V_6_As_8_O_26_]^4−^. The calculated gap energies at B3LYP/TZP level of the latter As-POVs is in the range of 3.5–4.2 eV. Therefore, considering Hoffmann's justifications, any new viable model of [V_10_As_12_O_40_]^4−^ would be expected to exhibit an energy gap within the same range or higher. To derive such a structure one may be tempted to take a saturated model of [V_12_As_12_O_42_] (see Fig. S1†) and then systematically to remove [VO]^2+^ cations towards derivation of [V_10_As_12_O_40_]^4−^ models. Although this method is very inefficient, the performed geometric optimizations showed that the set of derived structural models are unsuitable as they showed different levels of fragmentation, dangling moieties and inconsistent local binding configurations.

**Fig. 12:**
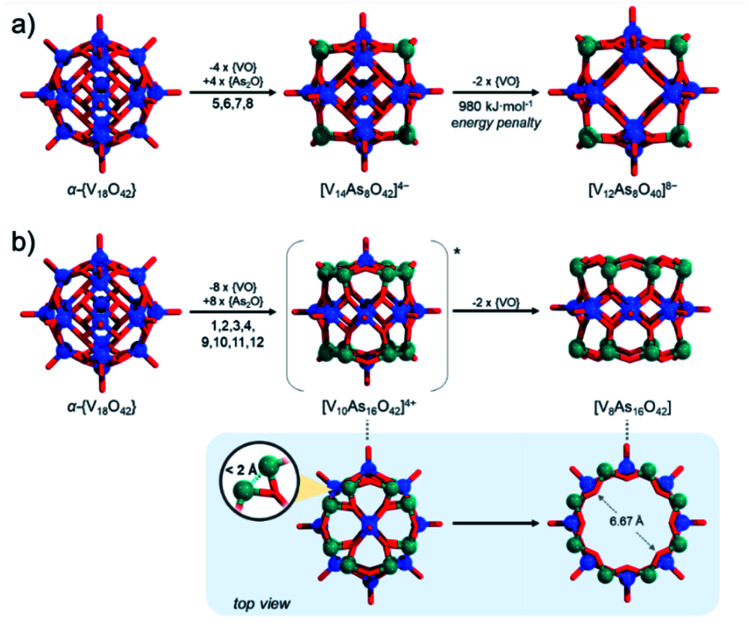
Schematic juxtaposition of (a) substitution of four [VO]^2+^ sites in the central belt of α-{V_18_O_42_} leading to energetically unfavourable further 2 × [VO]^2+^ loss; (b) substitution of eight [VO]^2+^ sites of the two cupolaed in α-{V_18_O_42_} leading to geometrically favourable 2 × [VO]^2+^ loss. Colour code: V = blue, As = green, and O = red spheres.

These issues with model derivation are not surprising. In the introduction, we mentioned the formation of unsaturated and mixed-valent [V_12_As_8_O_40_]^4−^ polyanions. In fact, formation of fully-reduced [V_12_As_8_O_40_]^8−^ by formal removal of two [VO]^2+^ from β-[V_14_As_8_O_42_]^4−^, by our estimations is not an enthalpically favourable process which may be a reason why mixed-valent species are isolated experimentally. Simple reaction models such as β-[V_14_As_8_O_42_]^4−^ + 8H_2_O → [V_12_As_8_O_40_]^8−^ + 2[VO(OH_2_)_4_]^2+^ (Fig. 12a) show that the reactants are some 980 kJ mol^−1^ lower in energy than the products formed. Furthermore, the fully reduced [V_12_As_8_O_40_]^8−^ cluster shows a HOMO–LUMO gap of 2.16 eV, which is significantly lower than any other known fully reduced As-POV as calculated at the same theoretical level. This result indicates that the derivation of the [V_10_As_12_O_40_]^4−^ model should occur in a way that the substitution pattern leads to steric and/or energetic changes that make the expulsion of two [VO]^2+^ cations desirable.

To generate β-[V_14_As_8_O_42_]^4−^ from α-{V_18_O_42_} we mentioned that one applies {VO} with {As_2_O} substitution at sites [1,3,9,11] which is virtually equivalent to substitution at [5,6,7,8] sites. Reversed pattern substitution implies that instead of the four [5,6,7,8] sites, the substitution takes place on the remaining eight sites, that is [1,2,3,4,9,10,11,12]. Such major substitutions would formally lead to a *D*_4h_ symmetric structure with the formula [V_10_As_16_O_42_]^4+^ (Fig. 12b). Modelling of [V_10_As_16_O_42_]^4+^ shows that the arsenate hetero-groups come very close to each other, having interatomic As^3+^⋯As^3+^ distances between neighbouring pairs of less than 2 Å. Such positioning of the As^3+^ centres is expected to cause charge and orbital repulsions. These repulsions lead to rearrangements that may no longer retain the bonding to the axial [VO]^2+^ cations, leading to the dissociation of the latter. Indeed, modelling of tubular and neutral [V_8_As_16_O_40_] shows that the cyclic species are geometrically reasonable and exhibit a calculated HOMO–LUMO gap energy of 4.5 eV, which is the highest among all known computationally studied As-POVs. The optimised structure increase the distances between the As^3+^ pairs, but it also changes the distances between the oxo-ligands binding the [VO]^2+^ cations in *trans*-fashion to 6.67 Å, making reversed binding and saturation with [VO]^2+^ unfavourable (see ESI Video†).

The tubular [V_8_As_16_O_40_] moiety can be similarly derived from the β-{V_18_O_42_} cluster shell, while its derivation principles outline a new abstract property for substitution in molecular kegginoids. Although [V_8_As_16_O_40_] appears to be a viably stable material in terms of gap energies owing to its neutral charge, it may be challenging to synthesise in polar solvents in its native form and thus it would also be undetectable by ESI-MS. However, [V_8_As_16_O_40_] demonstrates the interconnectedness between structural archetypes and provides a platform for how to derive a reasonable model set for [V_10_As_12_O_40_]^4−^ polyanions.

**Fig. 13:**
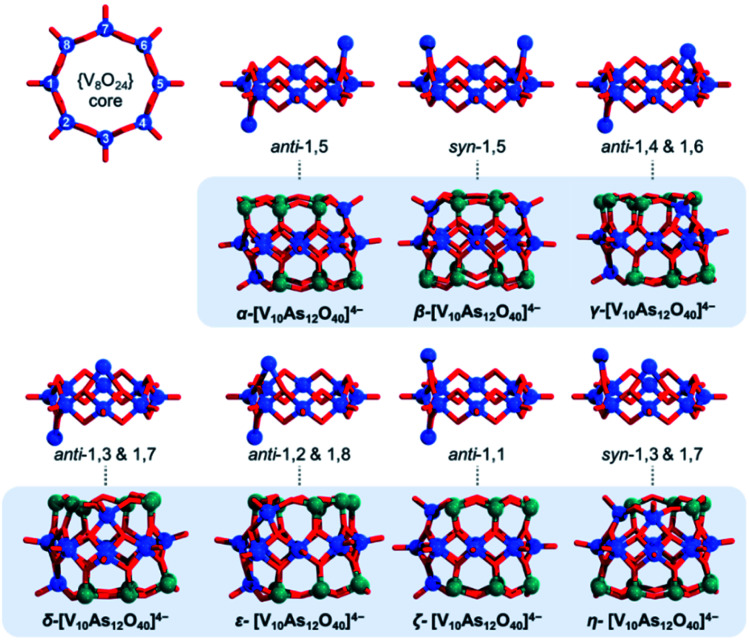
Enumeration of the eight V centres in the {V_8_O_24_} cores deriving from the [V_8_As_16_O_40_] structure (top left), followed by the depiction of substitution patterns and the respective [V_10_As_12_O_40_]^4−^ isomers positioned in the shaded box areas colour code: V = blue, O = red, and As = green spheres.

The tubular [V_8_As_16_O_40_] structure consists of a {V_8_O_24_} belt sandwiched between two {As_8_O_8_} rings. By substitution of two {As_2_O} units with two {VO}, one can formally derive seven configurational [V_10_As_12_O_40_]^4−^ isomers out of which four have enantiomeric pairs. To distinguish between the isomers, one can enumerate the eight V centres constituting the {V_8_O_24_} rings and can further specify whether the substitution occurs on the same side (*i.e. syn*) or on the opposite side (*i.e. anti*) of the {V_8_O_24_} rings (Fig. 12b). Such nomenclature helps in keeping track between isomers; however, it is also important in some way to indicate the relative distancing between substituting sites. To showcase the latter, one way would be to apply Greek alphabet letters α, β, γ, δ, ε, ζ, and η (Fig. 13), which follow the order in gradual interatomic distance decrease between the substituting V pairs (*i.e. d*(V⋯V)). Thus, in the optimized structures, the *d*(V⋯V) distances are in the range of 8.97 down to 3.54 Å. Although symmetric arrangement was considered prior to all calculations, the geometric optimization also showed that the structures that do not have enantiomeric analogues adopt highest symmetries (*i.e. anti*-1,5 = *C*_2h_, *syn*-1,5 = *C*_2v_ and *anti*-1,1 = *C*_2v_).

**Fig. 14:**
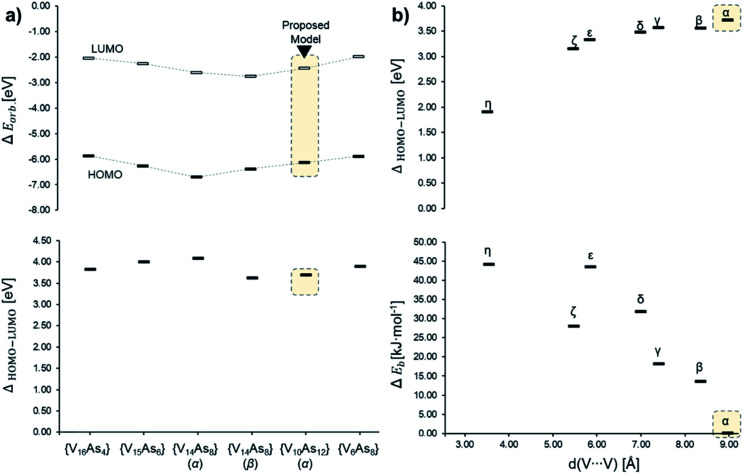
Energy diagrams based on Tables S7 and S8,† showcasing (a) the α-spin HOMO and LUMO (top) and HOMO–LUMO gap (bottom) energies among different experimentally described As-POVs and the herein proposed α-[V_10_As_12_O_40_]^4−^ model; (b) HOMO–LUMO gap (top) and relative bonding energy in of the isomeric [V_10_As_12_O_40_]^4−^ in comparison to the α-structure.

In the [V_10_As_12_O_40_]^4−^ series, the HOMO–LUMO energy gap shows a trend that is increasing with the distance between the substitution centres (Fig. 14). This implies that the highest gap energy of 3.7 eV is observed for the tubular α-[V_10_As_12_O_40_]^4−^ structure, while the lowest gap energy of 1.9 eV is calculated for η-[V_10_As_12_O_40_]^4−^. The bonding energy follows a similar trend; however, it shows an anomaly for the ζ-[V_10_As_12_O_40_]^4−^ which is energetically lower than the η- and ε-isomers. This energetic anomaly may derive from the fact that the *C*_2v_ symmetric ζ-[V_10_As_12_O_40_]^4−^ allows more uniform structural arrangement enhancing orbital overlaps. The energy difference between α-[V_10_As_12_O_40_]^4−^ and β-[V_10_As_12_O_40_]^4−^ is some 14 kJ mol^−1^. The structure of β-[V_10_As_12_O_40_]^4−^ is also highly symmetrical, and it resembles the semi-tubular isopolyoxovanadate isomer, [V_12_O_32_]^4−^ (see Fig. S1†). The least stable isomer η-[V_10_As_12_O_40_]^4−^ is some 44.2 kJ mol^−1^ higher in energy in comparison to α-[V_10_As_12_O_40_]^4−^.

The tubular isomers show relatively neat conversion; however, some may have a relatively flat geometric optimization route. This is because the tubular structures may be a subject of conformational isomerism transitioning from cyclic to different degrees of ellipsoidal arrangement of the polyoxovanadate belts. The phenomenon is also reflected in the IR spectrum (Fig. S16†), which does not show negative absorptions but some relatively low absorptions in the region between 0 and 100 cm^−1^ owing to lateral movement of the tube's walls.

**Fig. 15:**
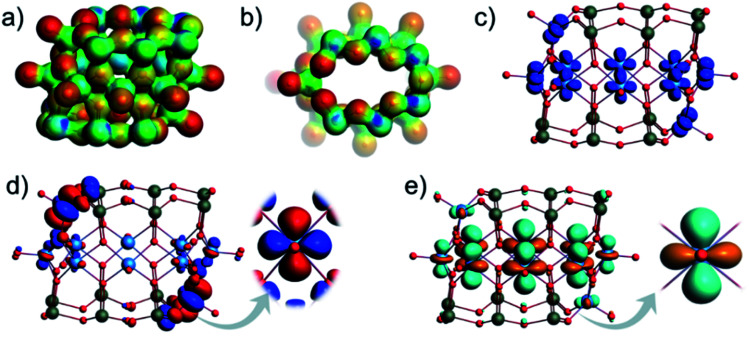
Molecular electrostatic potential (MEP) plotted over the density isosurface of [V_10_As_12_O_40_]^4−^ shown in (a) side and (b) top view. The most negative potentials are shown in red. Spin density isosurfaces indicating accumulation of α spins for [V_10_As_12_O_40_]^4−^ (c). Depiction of the HOMO (d) and the LUMO of [V_10_As_12_O_40_]^4−^ (e). Colour code: V = blue, O = red, and As = green spheres.

The optimization of [V_10_As_12_O_40_]^4−^ (*C*_2h_) structure showed that the calculated terminal V–O, bridging V–O, and As–O bond lengths fit the expectations for experimental ranges and are comparable to those of the other experimentally confirmed As-POVs as calculated at the same theoretical level (Table S6†).^25,76^ In α-[V_10_As_12_O_40_]^4−^, there are four inequivalent V centres and three inequivalent As centres (Fig. S14a†). The antiparallel arrangement of the outer {VAs_6_O_8_} rings enforces the ellipsoidal conformation of the {V_8_O_24_} ring. From the projection of the molecular electrostatic potential over the density isosurface (Fig. 15a and 14b), it is clear that bridging oxo ligands of the outer-ring V atoms exhibit the most negative potentials and thus exhibit the most nucleophilic sites. However, the nucleophilicity gradually decreases among the other bridging oxo ligands in the outer rings depicting an environment that is not suitable for polydentate binding of vanadyl cations. The spin density isosurface shows d-type atomic-like orbitals centred on each V atom, which is consistent with the expectation that all vanadium centres are in the +IV oxidation state (Fig. 15c). The calculated HOMO–LUMO gap of 3.7 eV compares well to the other experimentally characterized and theoretically described hetero-POVs (Fig. 14a).^25,76^ The HOMO depicts d_*xy*_ atomic-like orbitals centred on two outer vanadium atoms (Fig. 15d), while the LUMO depicts d_*z*_^2^ atomic-like orbitals delocalized on the V centres of the ellipsoidal {V_8_O_24_} ring (Fig. 15e). The overall transition implies a shift of electron density from the outer to the inner ring as a form of intramolecular d–d transitions. Infrared spectrum calculation does not show any negative frequencies (Fig. S16†), while the positioning of the most characteristic absorptions is in line with previous studies.^25^ All of the aspects taken together indicate the proposed [V_10_As_12_O_40_]^4−^ (*C*_2h_) structure to be a good model candidate for the structure of the ion observed by ESI-MS.

## 4 Conclusions

The conclusions section should come in this section at the end of the article, before the acknowledgements.

The current work is a product of applied insights and experience that we have gained after many years of dedicated effort in synthetic, analytical and computational POV chemistry. The interest in kegginoidal {V_18−*x*_(As_2_)O_42_} architectures has been of focal interest for the past three decades, *e.g.*, as it directly relates to the design of magnetically responsive As-POVs. Using the overlooked system [V_6_As_8_O_26_]^4−^, we carefully designed water-soluble species that enabled solution ageing studies. We made the astonishing observation that ageing an aqueous solution of [V_6_As_8_O_26_]^4−^ at room temperature affords crystallization of the mixed-valent high-nuclearity [V_12_As_8_O_40_(H_2_O)]^4−^ anion, where the number of V centres is doubled compared to the starting sample. This is in complete contrast to that what is known in POM chemistry, where larger clusters usually fragment into smaller units. The observation made here implies that smaller fragments present in solution self-assembly to form the final cluster shell. Thorough time-dependent ESI-MS studies evidenced a slow disappearance of the mass of the small cluster anion, while a larger mass continuously grown, which could not be assigned to any structure of reported As-POV clusters. The new mass was assigned to an As-POV with composition [V_10_As_12_O_40_]^4−^. The grand challenge for identifying a possible structure was the huge number of possible structural configurations. Using the apparatus of mathematical stereochemistry, we successfully modelled the substitution group of {V_18−*x*_(As_2_)O_42_} and enumerated the thousands of possible configurations. However, with the constraints enforced by the empirical discovery of [V_10_As_12_O_40_]^4−^ and the guidelines on realistic molecular modelling, we deduced the configurational intertwining of the hetero-group substitution and the {VO}-cationic (de)stabilisation, which subsequently led to the rational design of a set of tubular molecular models. The devised structure shows a clear resemblance between kegginoidal (*i.e.* closed cage-like) archetypes and tubular (*i.e.* cyclic archetypes). It further provides insight that particular substituting configurations can trigger an increase in intramolecular strains and programmable archetype dynamics, which is valuable for further solution studies of POM speciation and design of new POM architectures.

The presented results in this article are also a by-product of a more holistic, *i.e.*, systems thinking approach. To reveal the interdependency in an extensive configurational system, we had to solve many interrelated problems, connecting different chemicals, concepts and techniques. Currently, the systems approach in chemistry is rarely applied; however, it is a key approach in solving challenging problems and, thus, is of increasing demand in the chemistry curriculum.^110,111^ In chemistry, the systems thinking approach is innate in domains that deal with networks of interacting molecules (*e.g.* systems chemistry),^112–115^ or alternatively networks of formally described chemical knowledge and information (*e.g.* digital chemistry).^116–118^ With the latter in mind, the present work unveils that POM do not exist as isolated instances, and on the contrary, they form large graphs of species, each having its unique attributes while connecting relationally to other species and concepts. In that context, the present work shows an example on how to navigate through such a complexity, and thus it can also serve as a promising approach for resolving similar problems in POM chemistry and cluster science.

## Data availability

Crystallographic data for compounds 1 and 2 has been deposited at the CCDC under 2128841 and 2130016. The characterisation and computational data, including optimised geometries supporting this article have been uploaded as part of the ESI.† The code for generation of POV isomers can be found at the git repository under https://www.github.com/digital-chemistry/pov_isomeriser.

## Author contributions

Conceptualisation A. K., C. A. S. and W. B.; single crystal X-ray diffraction and structure determination C. N.; structure solution and refinement from X-ray powder diffraction data S. M.; *in situ* study on conductivity and pH N. P. and M. R.; synthesis and characterization M. R.; ESI-MS D. S. and C. A. S.; software, V. S.; DFT calculations A. K., writing – original draft preparation, A. K.; writing – review and editing, A. K., C. A. S.,W. B., D. S., C. N., S. M., V. S., and N. P.; project administration, W. B.; funding acquisition, C. A. S. and W. B.; all authors have read and agreed to the published version of the manuscript.

## Conflicts of interest

There are no conflicts to declare.

## Supplementary Material

SC-013-D2SC01004F-s001

SC-013-D2SC01004F-s002

SC-013-D2SC01004F-s003

SC-013-D2SC01004F-s004
