# Supplementary material for: Composition-driven archetype dynamics in polyoxovanadates
Source: Chem Sci. 2022 Apr 29;13(21):6397–412. doi: 10.1039/d2sc01004f (PMC9159092; doi:10.1039/d2sc01004f)
Supplement: SC-013-D2SC01004F-s001 [file SC-013-D2SC01004F-s001.pdf]

Electronic Supplementary Material (ESI) for Chemical Science.
This journal is © The Royal Society of Chemistry 2022

Supporting Information

Composition-driven Archetype Dynamics in Polyoxovanadates

Aleksandar Kondinski,[a*] Maren Rasmussen,[b] Sebastian Mangelsen,[b] Nicole Pienack,[b] Viktor Simjanoski,[c] Christian Näther,[b] Daniel L. Stares,[d] Christoph A. Schalley,[d*] Wolfgang Bensch[b*]

a) Department of Chemical Engineering and Biotechnology, University of Cambridge, Philippa Fawcett Drive S, CB3 0AS, United Kingdom. E-mail: aleksandar@kondinski.com
b) Institut für Anorganische Chemie, Christian-Albrechts-Universität zu Kiel, 24118 Kiel, Germany. E-mail: wbensch@ac.uni-kiel.de
c) Primer affiliate of University of Chicago Master Program, Chicago, IL, United States.
d) Institut für Chemie und Biochemie der Freien Universität, Arnimallee 20, 14195 Berlin, Germany. E-mail: c.schalley@fu-berlin.de

Chemical Characterisation

The X-ray powder diffraction pattern of $[Ni(en)_3]_2[V_6As_8O_{26}]$ was collected with a STOE Stadi P diffractometer (STOE & Cie) equipped with a MYTHEN 1 K detector (DECTRIS) using germanium monochromatized Cu-$K_{\alpha 1}$ radiation (λ= 1.540598 Å). The experimental and the calculated patterns of **I** are displayed in Figure S2 demonstrating phase purity of the reaction product.
Energy dispersive X-ray spectroscopy (EDX) analyses were performed with a Philips environmental scanning electron microscope ESEM XL30 equipped with an EDX detector. Experimental data for the Ni:As:V ratio = 1.96 : 7.88 : 6.02.
CHN elemental analyses were done with a "vario MICRO Cube" instrument (Elementar Analysensysteme GmbH). Experimental data/expected data in %: N 9.21/9.34; C 8.04/8.01; H 2.81/2.69.
UV/Vis measurements were done on a two-channel Cary 5 spectrometer (Varian Techtron Pty., Darmstadt, 200–3000 cm^{-1}). The solid material was ground with $BaSO_4$ as white standard and the data are presented as Kubelka-Munk relation for diffuse reflectance data (Figure S3). Aqueous solutions (5 mL) at different concentrations were measured in quartz cuvettes and the maximum solubility was determined using the intense absorption at 610 nm (Figure S4).
The IR spectrum was recorded using an ATI Mattson Genesis Series FTIR Spectrometer, control software: WINFIRST, from ATI Mattson (Figure S5).

The long-term stability experiments were carried out with the synthesis workstation EasyMax® 102 (Mettler Toledo). Several sensors/electrodes were adapted by using a universal control box (UCB). The operation was done by the iControl EasyMax® software (Mettler Toledo). For the experiment, 594.5 mg $[Ni(en)_3]_2[V_6As_8O_{26}]$ were dissolved in 40 mL bi dest. water (≙ 14.86 g/L ≙ 8.26 mmol/L) in a 50 mL glass reactor at T = 25 °C, stirred with r = 500 rpm and equipped with pH electrodes and conductivity sensor. The temperature was kept at 25°C for twelve days monitoring the changes in pH value and ionic conductivity. The pH sensors (InLab 1022 resp. Semi Micro L, Mettler Toledo) are combined pH electrodes with the following data: pH range 1-14, temperature range 0-80°C resp. 0-100°C, ARGENTHAL™ reference system, ceramic reference diaphragm, 3 mol/L KCl reference electrolyte. The ionic conductivity sensor (LTG Zelle, Sensortechnik Meinsberg, Xylem Analytics) is a conductivity cell for measurements in ionic mediums with 2-electrode technology, platinum electrodes (3·4 mm with a distance of 2 mm) and integrated temperature sensor (cell constant 1 cm^{-1}).

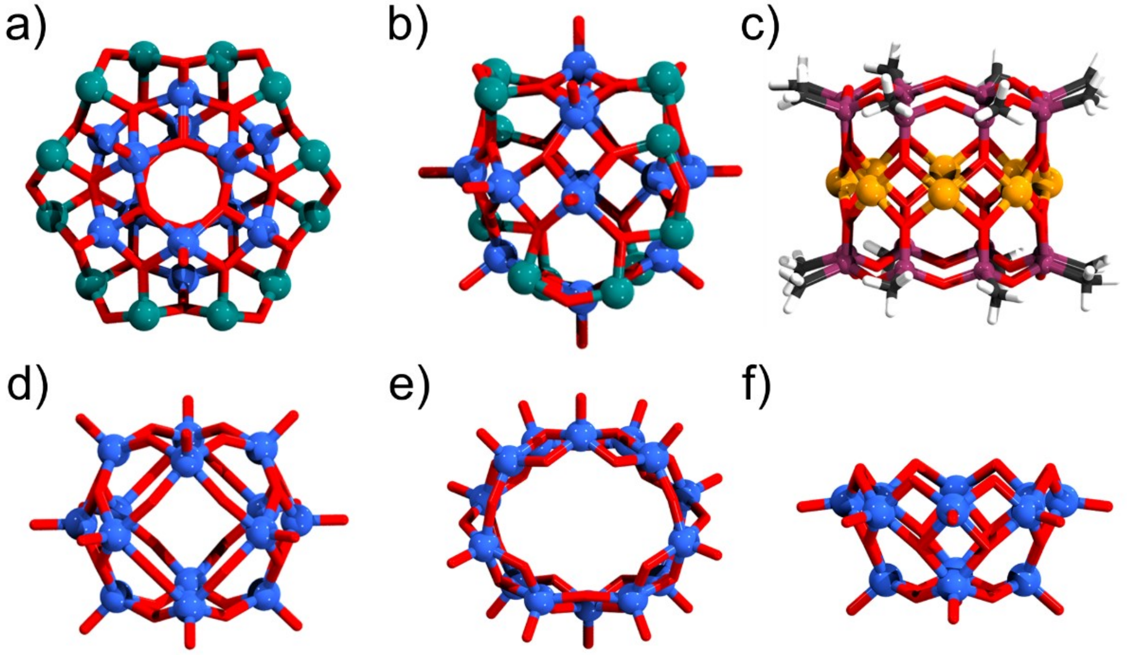

Figure S1: Ball and stick representation of: a) D_{3d} symmetric $[V_{12}O_{36}(As_2O)_6]$ b) D_2 symmetric $[V_{12}O_{36}(As_2O)_6]$; e) cyclic $\{Cu_8O_{16}(CH_3SiO)_8\}$; d) unsaturated kegginoidal $\{V_{14}O_{38}\}$. e) tubular $\{V_{14}O_{32}\}$; f) $[V_{12}O_{32}]^{4-}$ bowl. Colour code: V = blue, Cu = orange, As = green, O = red, Si = magenta, C = black and H = white.

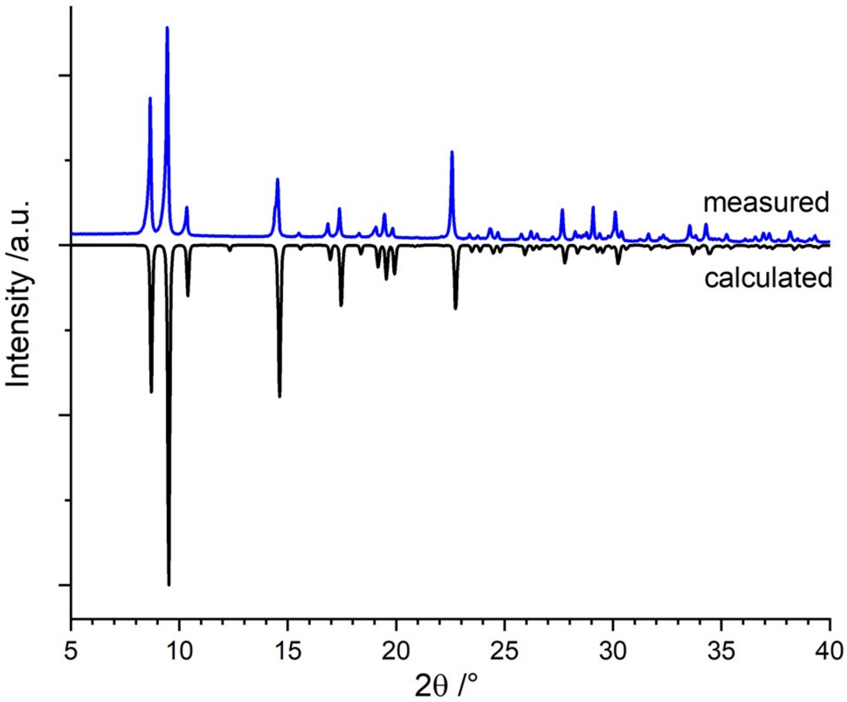

Figure S2: Comparison of the measured and calculated X-ray powder patterns of $[Ni(en)_3]_2[V_6As_8O_{26}]$.

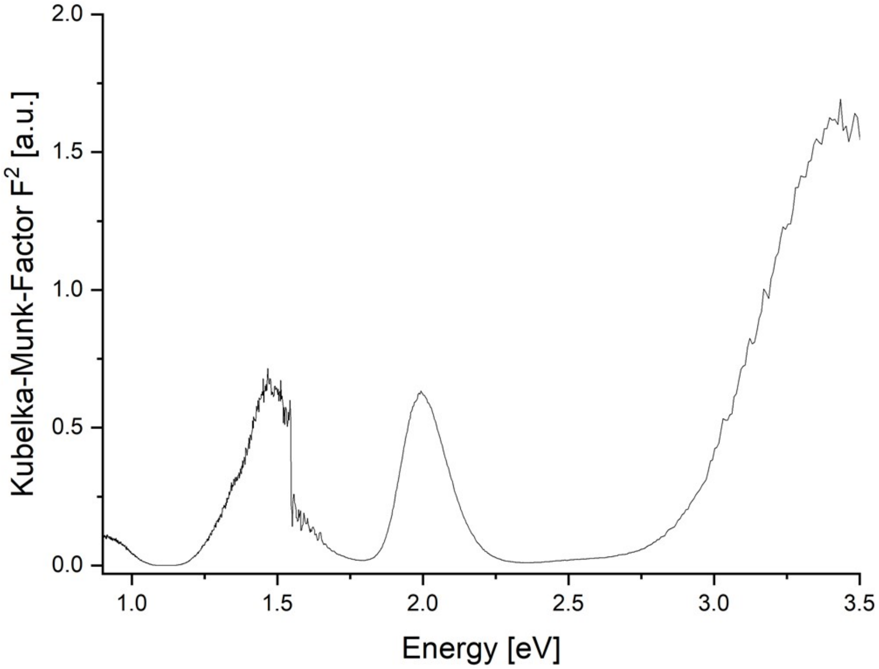

Figure S3: UV-Vis spectrum of $[Ni(en)_3]_2[V_6As_8O_{26}]$ shown as Kubelka-Munk plot. First absorption at ca. 1.45 eV (11700 cm^{-1}, 855 nm), second at 2 eV (16130 cm^{-1}, 620 nm). The intense absorption above 3 eV is caused by a charge-transfer transition.

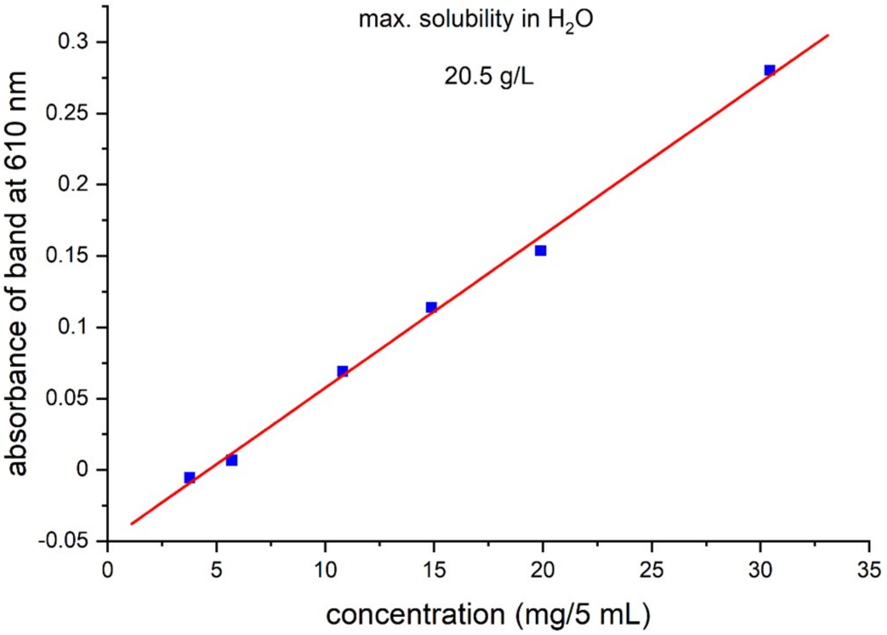

Figure S4: Determination of the maximum solubility of $[Ni(en)_3]_2[V_6As_8O_{26}]$ in water using the absorption at 610 nm in the UV/Vis spectra.

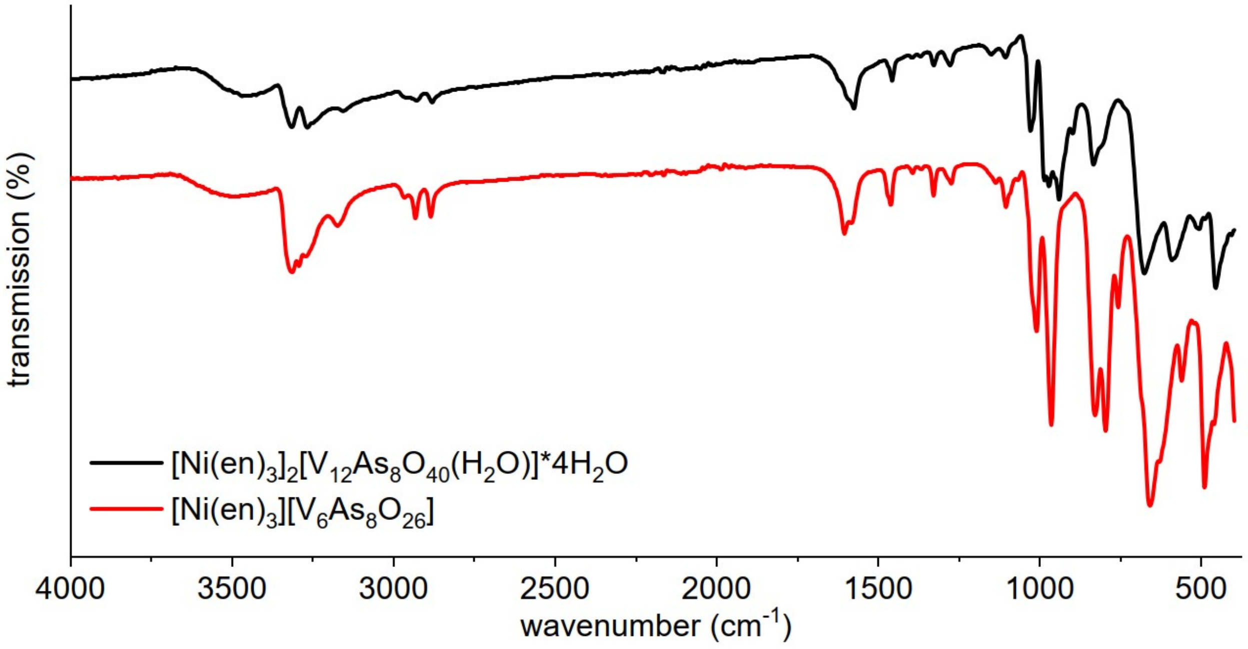

Figure S5. Comparison of the IR spectra for compound **1** (red) and **2** (black). Note the substantial changes in the vibrations in the range of 1100 – 600 cm^{-1}, which are mainly due to metal-oxygen modes. Assignments: 3450 cm^{-1} (O-H stretch), 3315 – 3150 cm^{-1} (N-H stretching), 2970-2880 cm^{-1} (C-H stretching), 1600-1580 cm^{-1} (-NH_2 bending), 1150-1100 cm^{-1} (CH_2 bending/deformation), ~ 960 cm^{-1} (terminal V=O), ~670 cm^{-1} (V-O-V).[1,2]

Table S1. Selected crystal data and results of the structure refinement.

**compound**	**1**
Formula	$C_{12}H_{48}As_8N_{12}Ni_2O_{26}V_6$
MW / g mol^{-1}	1798.9797
crystal system	tetragonal
space group	$I4_1/acd$
a / Å	20.3058(5)
b / Å	20.3058(5)
c / Å	24.3847(7)
V / Å^3	10054.4(6)
T / K	200(2)
Z	8
D_{calc} / g cm^{-3}	2.377
μ / mm^{-1}	7.109
θ_{max} / deg	26.004
measured refl.	33243
unique refl.	2477
refl. $F_0 > 4\sigma(F_0)$	2245
parameter	151
R_{int}	0.0854
R_1 [$F_0 > 4\sigma F_0$]	0.0396
wR_2 [all data]	0.0950
GOF	1.198
$\Delta\rho_{max/min}$ / e Å^{-3}	0.388/-0.438

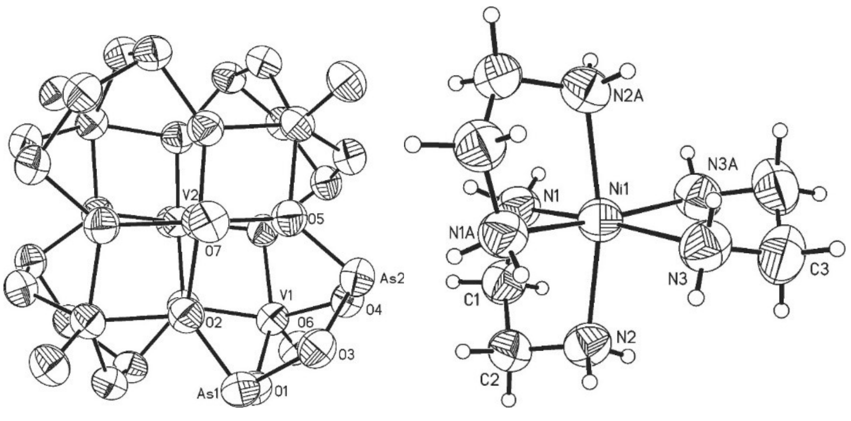

Figure S6. View of the cluster anion (left) and of the Ni^{2+} centred complex (right) with labelling and displacement ellipsoids drawn at the 50% probability level.

Details of the crystal structure determination of $[Ni(en)_3]_2[V_{12}As_8O_{40}(H_2O)]\cdot4H_2O$

The X-ray powder diffraction pattern did not coincide with that of $[Ni(en)_3]_2[V_6As_8O_{26}]$ and did not match with any pattern of As-POVs. All reflections of the XRPD pattern could be indexed successfully (monoclinic, $P2_1/n$) with lattice parameters a = 23.153 Å, b = 11.920 Å, c = 11.731 Å and β = 95.233°. The IR spectrum showed absorptions of the $[Ni(en)_3]^{2+}$ cation but significant differences in the region of As-O and V-O vibrations were evident compared to $[Ni(en)_3]_2[V_6As_8O_{26}]$ (see Figure S5). The refined sum formula of the compound is $[Ni(en)_3]_2[V_{12}As_8O_{40}(H_2O)]\cdot4H_2O$, however, the water content should not be over interpreted since it is solely based on the residual electron density and not on precise chemical analytics. The unit cell content was estimated based on the available data: The $[Ni(en)_3]^{2+}$ complex and the ratio of the metals were included, the number of oxygen atoms was calculated to match the remaining unit cell volume. No information on the cluster type was available, which renders this case a particular difficult one. The intensity data exhibit outstanding sharp reflections due to the low instrumental contribution and excellent counting statistics. Applying the charge flipping method resulted in the location of numerous metal ions in a cluster-like motif and some of the oxygen atoms were located at reasonable positions as well. This starting model was subsequently improved in a Rietveld refinement guided by chemical knowledge. First, all atoms not attached to the cluster were removed and the $[Ni(en)_3]^{2+}$ cation was introduced as rigid body with only rotational and translational degrees of freedom. The initial position was determined by simulated annealing and further subjected to unconstrained movement in the further steps. Next, the cluster motif was refined assuming that structural building blocks like e.g. VO_5 pyramids and As_2O_5 dumbbells are intact. The VO_5 pyramids are treated as rigid bodies leading to a drop of the R_{wp} value, while the As atoms were refined individually, since the connection to oxygen atoms is determined by the attached VO_5 rigid bodies. Further refinements and continuous inspection of the Fourier maps led to identification of the $[V_{12}As_8]$ cluster type. At this stage the structure solution was restarted in real space with large rigid bodies significantly reducing the number of refined parameters. The $[V_{12}As_8O_{40}]^{4-}$ ion has a fourfold rotational symmetry and in the space group $P2_1$/n half of it is symmetry generated. Two identical rigid bodies comprising $As_2V_3O_{10}$ together with a rigid body representing the $[Ni(en)_3]^{2+}$ cation were subjected to global optimisation by simulated annealing. A very similar result to the above described procedure was obtained and subjected to a Rietveld refinement after solving the structure. By inspection of the Fourier map additional electron density was found that could be assigned to water molecules: one at a special position in the centre of the cluster, which is often observed in related As-POVs,[2,3] and two additional which are most probable of H_2O molecules. The presence of water is supported by the broad absorption centred at 3450 cm^{-1} in the IR spectrum (Figure S5). In a final Rietveld refinement the location of the rigid bodies and water molecules could be subjected to a stable refinement converging with a convincing R_{wp} of 4.7 %, the difference plot of the final fit is presented in the Figure 6 in the main part (in the Table below the data for the refinements is summarized). Further support for the validity of the identified structure can be derived comparing the structural features with those found in literature: $[NHEt_3]_4[V_{12}As_8O_{40}(H_2O)]\cdot H_2O$,[3] and $[NHEt_3]_2[NH_2Me_2][V_{12}As_8O_{40}(HCO_2)]\cdot2H_2O$,[4] are examples with the same cluster and large cations, that both crystallize in the space group $P2_1/c$. Interestingly, the corresponding ammonium salt of compound **2** crystallizes in the higher symmetric space group *Pnnm*.[2]

Table S2. Selected crystal data and results of the structure refinement for $[Ni(en)_3]_2[As_8V_{12}O_{40}(H_2O)]\cdot 4H_2O$

**Compound**	**2**
Formula	$C_{12}As_8N_{12}Ni_2O_{45}V_{12}$
MW / g mol^{-1}	2360.26
crystal system	monoclinic
space group	$P2_1/n$
a / Å	23.1578(5)
b / Å	11.9216(3)
c / Å	11.7372(4)
β / °	95.2333(16)
V / Å^3	3226.88(15)
T / K	265(2)
Z	2
D_{calc} / g cm^{-3}	2.429
μ / mm^{-1}	3.382
Wavelength / Å	0.5639132
R_p / %	4.03
R_{wp} / %	5.21
R_{exp} / %	0.47
R_{Bragg} / %	3.66
Starting angle / ° 2θ	1.5
Final angle / ° 2θ	32

Table S3. Bond lengths [Å] and angles [°] for the cluster anion. Symmetry transformations used to generate equivalent atoms: #1 -y+5/4,x+1/4,-z+3/4; #2 y-1/4,-x+5/4,-z+3/4; #3 -x+1,-y+3/2,z+0; #4 -y+5/4,-x+5/4,-z+5/4.

As(1)-O(1)	1.709(3)	As(2)-O(4)	1.726(3)
As(1)-O(3)	1.800(3)	As(2)-O(3)	1.792(3)
As(1)-O(2)	1.814(3)	As(2)-O(5)	1.795(3)
V(1)-O(6)	1.599(4)	V(2)-O(7)	1.602(5)
V(1)-O(1)	1.916(3)	V(2)-O(5)#3	1.960(3)
V(1)-O(4)	1.935(3)	V(2)-O(5)	1.960(3)
V(1)-O(2)#1	2.000(3)	V(2)-O(2)	1.960(3)
V(1)-O(5)#2	2.001(3)	V(2)-O(2)#3	1.960(3)
O(2)-V(1)#2	2.000(3)	O(5)-V(1)#1	2.001(3)
O(1)-As(1)-O(3)	102.79(16)	O(4)-As(2)-O(3)	101.36(16)
O(1)-As(1)-O(2)	100.94(16)	O(4)-As(2)-O(5)	101.46(15)
O(3)-As(1)-O(2)	95.36(15)	O(3)-As(2)-O(5)	96.66(15)
O(6)-V(1)-O(1)	106.59(17)	O(7)-V(2)-O(5)#3	108.02(10)
O(6)-V(1)-O(4)	105.43(17)	O(7)-V(2)-O(5)	108.02(10)
O(1)-V(1)-O(4)	89.64(15)	O(5)#3-V(2)-O(5)	144.0(2)
O(6)-V(1)-O(2)#1	107.57(16)	O(7)-V(2)-O(2)	109.16(10)
O(1)-V(1)-O(2)#1	145.09(14)	O(5)#3-V(2)-O(2)	76.75(14)
O(4)-V(1)-O(2)#1	88.18(14)	O(5)-V(2)-O(2)	91.49(14)
O(6)-V(1)-O(5)#2	107.85(17)	O(7)-V(2)-O(2)#3	109.16(10)
O(1)-V(1)-O(5)#2	87.99(14)	O(5)#3-V(2)-O(2)#3	91.49(14)
O(4)-V(1)-O(5)#2	145.88(14)	O(5)-V(2)-O(2)#3	76.75(14)
O(2)#1-V(1)-O(5)#2	74.92(14)	O(2)-V(2)-O(2)#3	141.7(2)
As(1)-O(1)-V(1)	128.53(19)	As(2)-O(4)-V(1)	128.52(18)
As(1)-O(2)-V(2)	132.55(18)	As(2)-O(5)-V(2)	131.87(18)
As(1)-O(2)-V(1)#2	121.92(17)	As(2)-O(5)-V(1)#1	123.12(17)
V(2)-O(2)-V(1)#2	104.05(15)	V(2)-O(5)-V(1)#1	104.02(15)
As(2)-O(3)-As(1)	126.92(19)		

Table S4. Bond lengths [Å] and angles [°] for the Ni coordination. Symmetry transformations used to generate equivalent atoms: A: -y+5/4,-x+5/4,-z+5/4

Ni(1)-N(1)	2.115(4)	Ni(1)-N(3)	2.125(4)
Ni(1)-N(1A)	2.115(4)	Ni(1)-N(2)	2.127(4)
Ni(1)-N(3A)	2.125(4)	Ni(1)-N(2A)	2.127(4)
N(1)-Ni(1)-N(1A)	94.9(3)	N(3A)-Ni(1)-N(2)	95.45(17)
N(1)-Ni(1)-N(3A)	92.61(18)	N(3)-Ni(1)-N(2)	93.64(17)
N(1A)-Ni(1)-N(3A)	170.55(18)	N(1)-Ni(1)-N(2A)	91.40(16)
N(1)-Ni(1)-N(3)	170.55(18)	N(1A)-Ni(1)-N(2A)	80.52(16)
N(1A)-Ni(1)-N(3)	92.61(18)	N(3A)-Ni(1)-N(2A)	93.64(17)
N(3A)-Ni(1)-N(3)	80.5(3)	N(3)-Ni(1)-N(2A)	95.44(17)
N(1)-Ni(1)-N(2)	80.52(16)	N(2)-Ni(1)-N(2A)	168.1(2)
N(1A)-Ni(1)-N(2)	91.40(16)		

Table S5. Hydrogen bonds [Å and °]. Symmetry transformations used to generate equivalent atoms: #1 -y+5/4,x+1/4,-z+3/4; #5 -x+1,-y+2,-z+1; #6 -x+1,y,z+1/2; #7 y-3/4,x+1/4,z+1/4.

D-H...A	d(D-H)	d(H...A)	d(D...A)	<(DHA)
N(1)-H(1N)...O(7)	0.91	2.16	2.975(5)	149.2
N(1)-H(2N)...O(6)#1	0.91	2.48	3.233(6)	139.9
N(2)-H(3N)...O(4)#5	0.91	2.16	3.034(5)	159.9
N(2)-H(4N)...O(6)#6	0.91	2.17	3.024(5)	156.5
N(3)-H(5N)...O(4)#7	0.91	2.31	3.191(6)	163.9
N(3)-H(6N)...O(6)#6	0.91	2.46	3.277(6)	148.8
C(3)-H(3A)...O(6)#5	0.99	2.63	3.616(7)	178.2

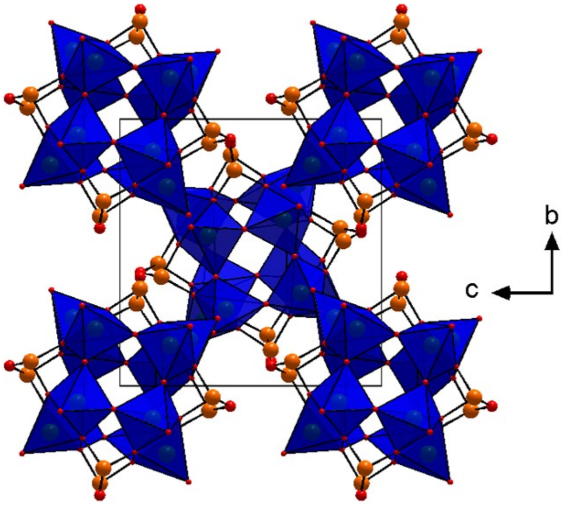

Figure S7. Arrangement of the cluster anions in the unit cell of the structure of compound **2**.

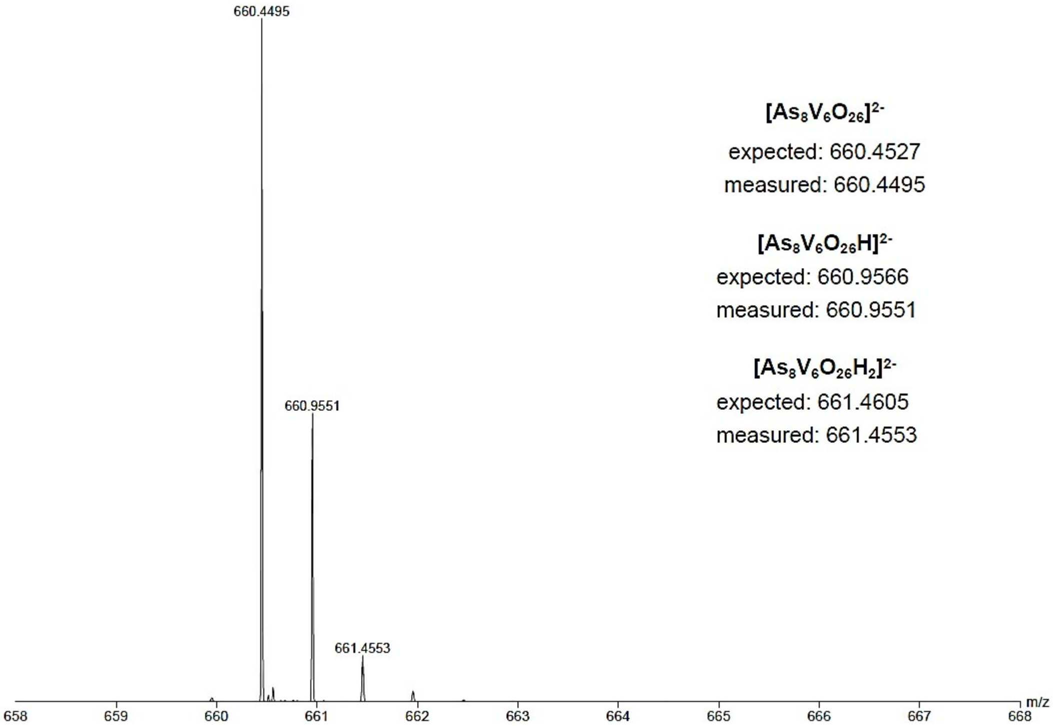

Figure S8: Zoom into the region containing the ion at *m/z* 660 of the initial measurement for the sample of the parent compound in H_2O (100 μM). Ionisation proceeds via loss of the counterions and subsequent double oxidation to produce -2 ions. Several different oxidation states are present with charge compensation coming from protonation.

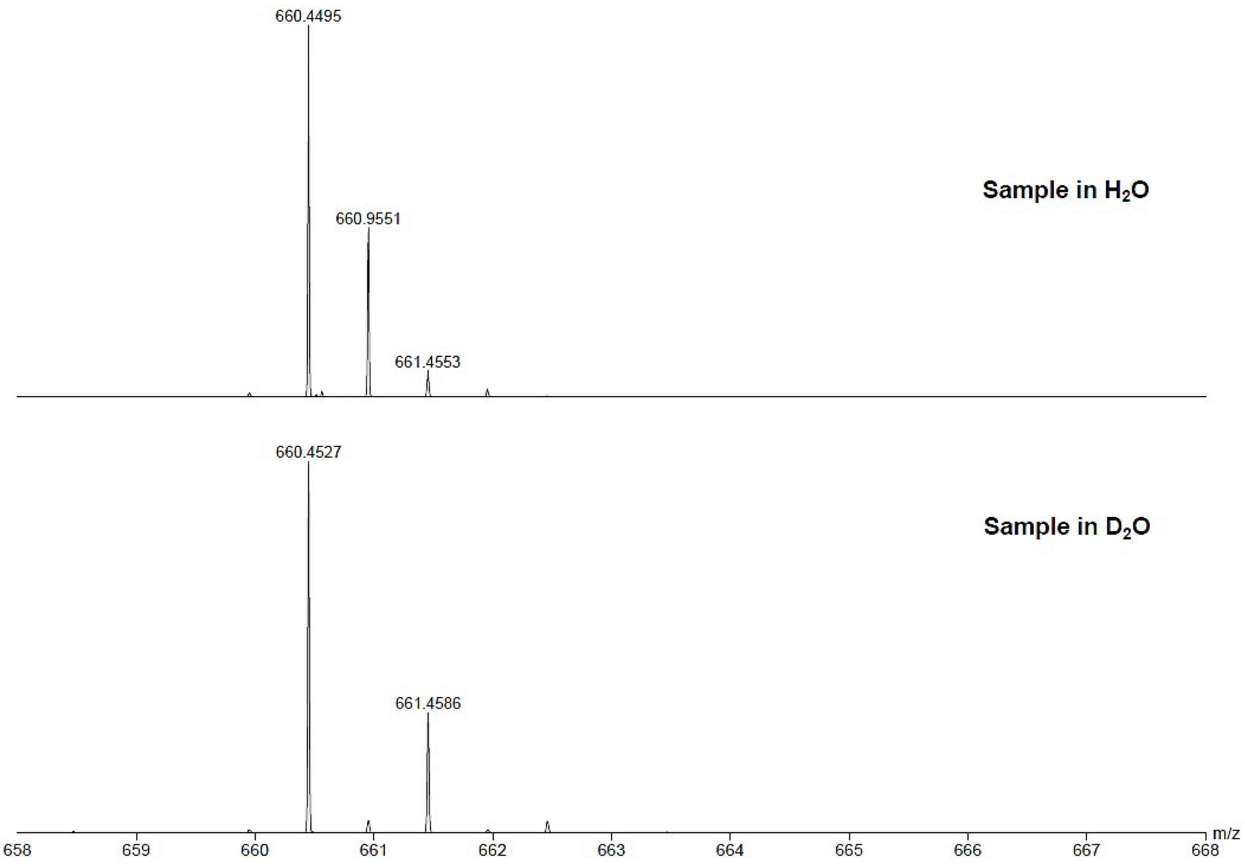

Figure S9: Initial measurements of 100 µM solution in H_2O (top) and in D_2O (bottom). As the elements in the cluster are almost completely monoisotopic, the change in peak spacing from 1 (upper spectrum; protonated) to 2 (bottom spectrum; deuterated) clearly confirms the assignment as protonated clusters appear as deuterated clusters in the bottom spectrum.

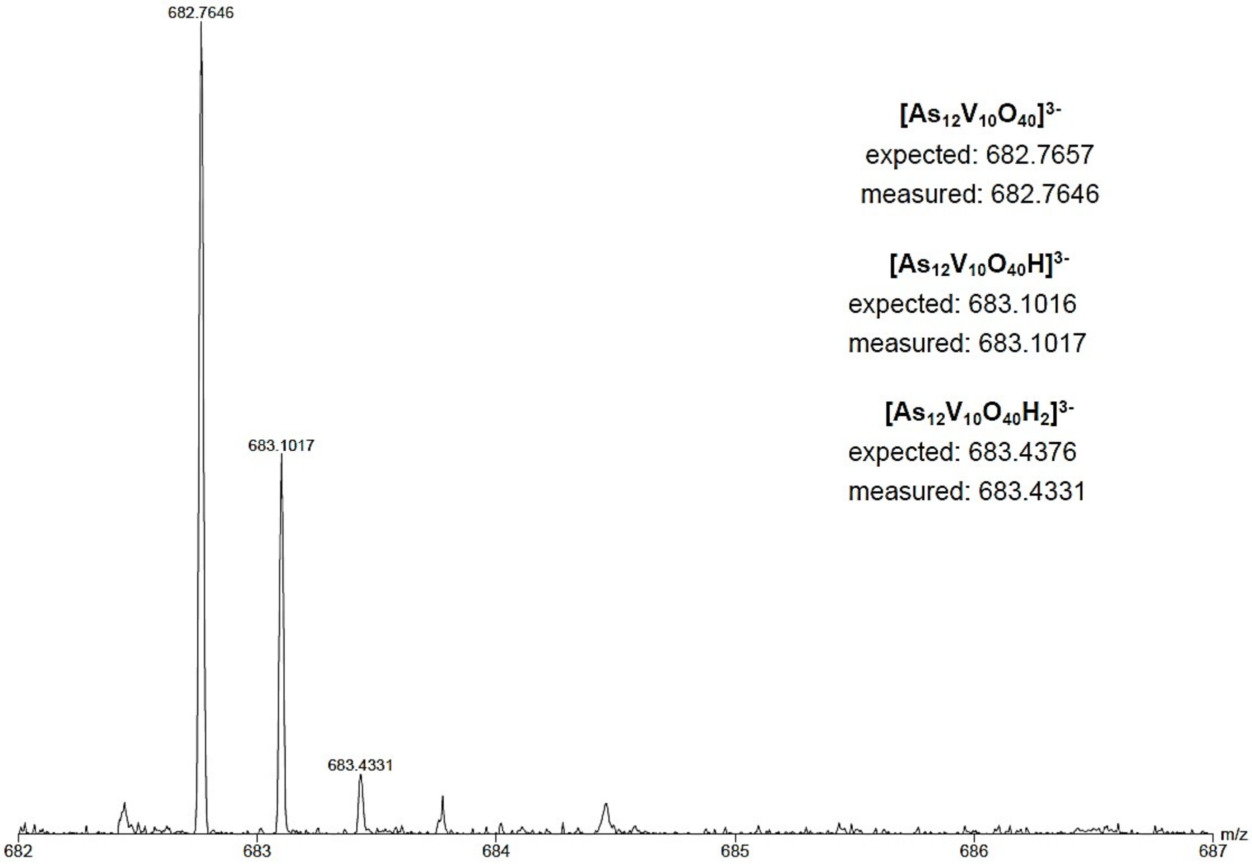

Figure S10: Zoom into the region containing the ion at *m/z* 682 of the sample in H_2O which is formed after several days. The signals and their spacing are consistent with the -3 charge state of a $[As_{12}V_{10}O_{40}]$ cluster. Several different oxidation states are present with charge compensating protons.

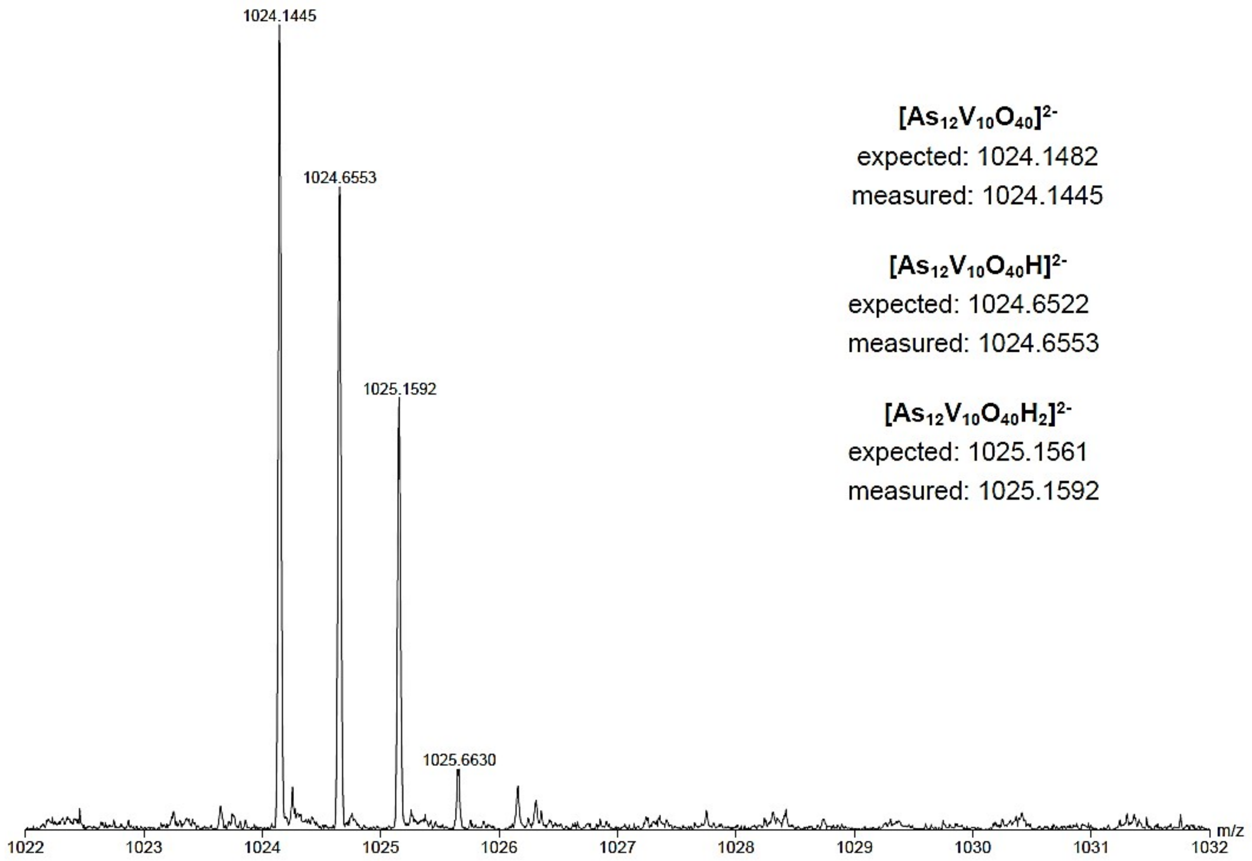

Figure S11: Zoom into the region containing the ion at *m/z* 1024 of the sample in H_2O which is formed after several days. The signals and their spacings are consistent with the -2 charge state of a $[As_{12}V_{10}O_{40}]$ cluster. Several different oxidation states are present with charge compensating protons.

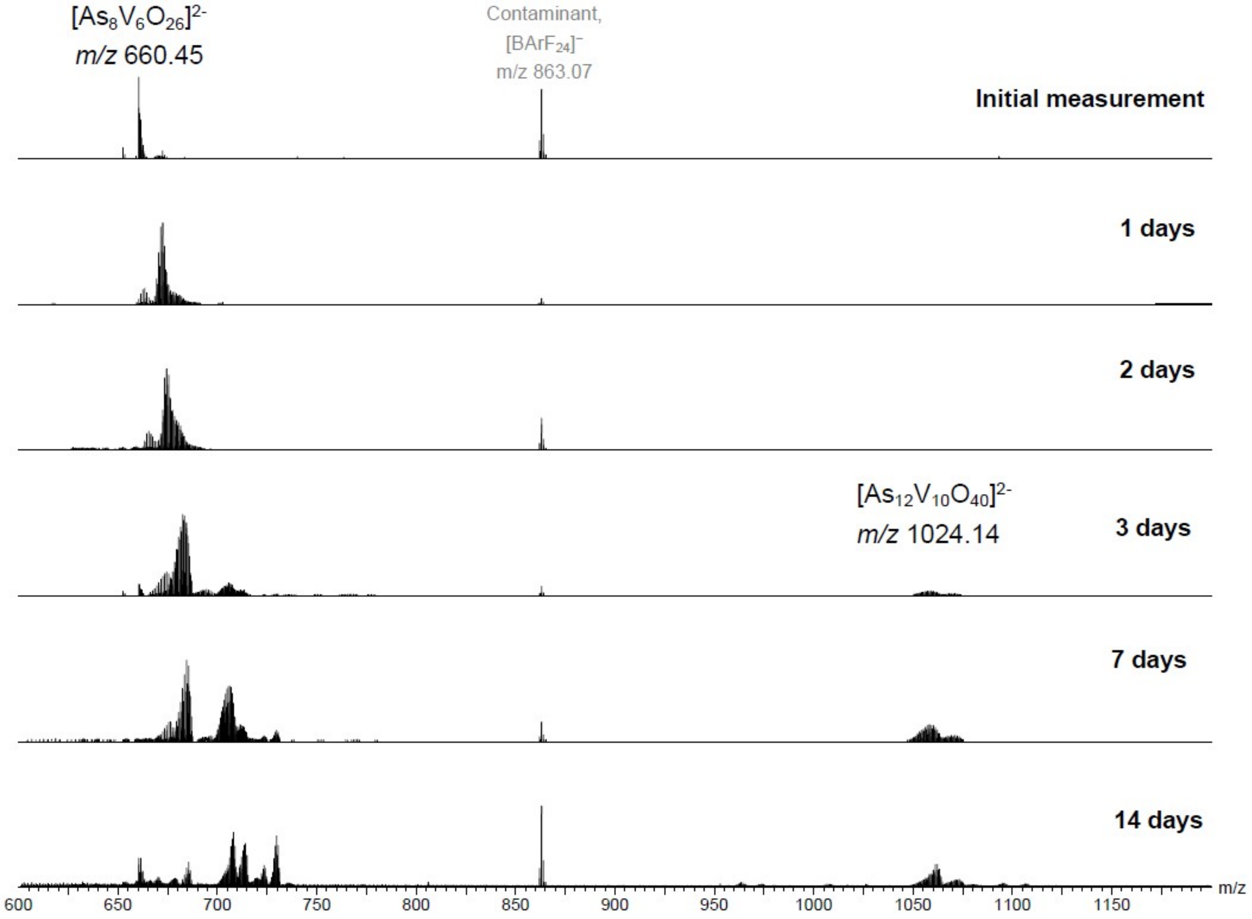

Figure S12: Full spectra of the samples in $H_2{}^{18}O$ measured after different time intervals. The cluster undergoes exchange over the course of a few days occurring faster than the loss of the initial and formation of the new cluster.

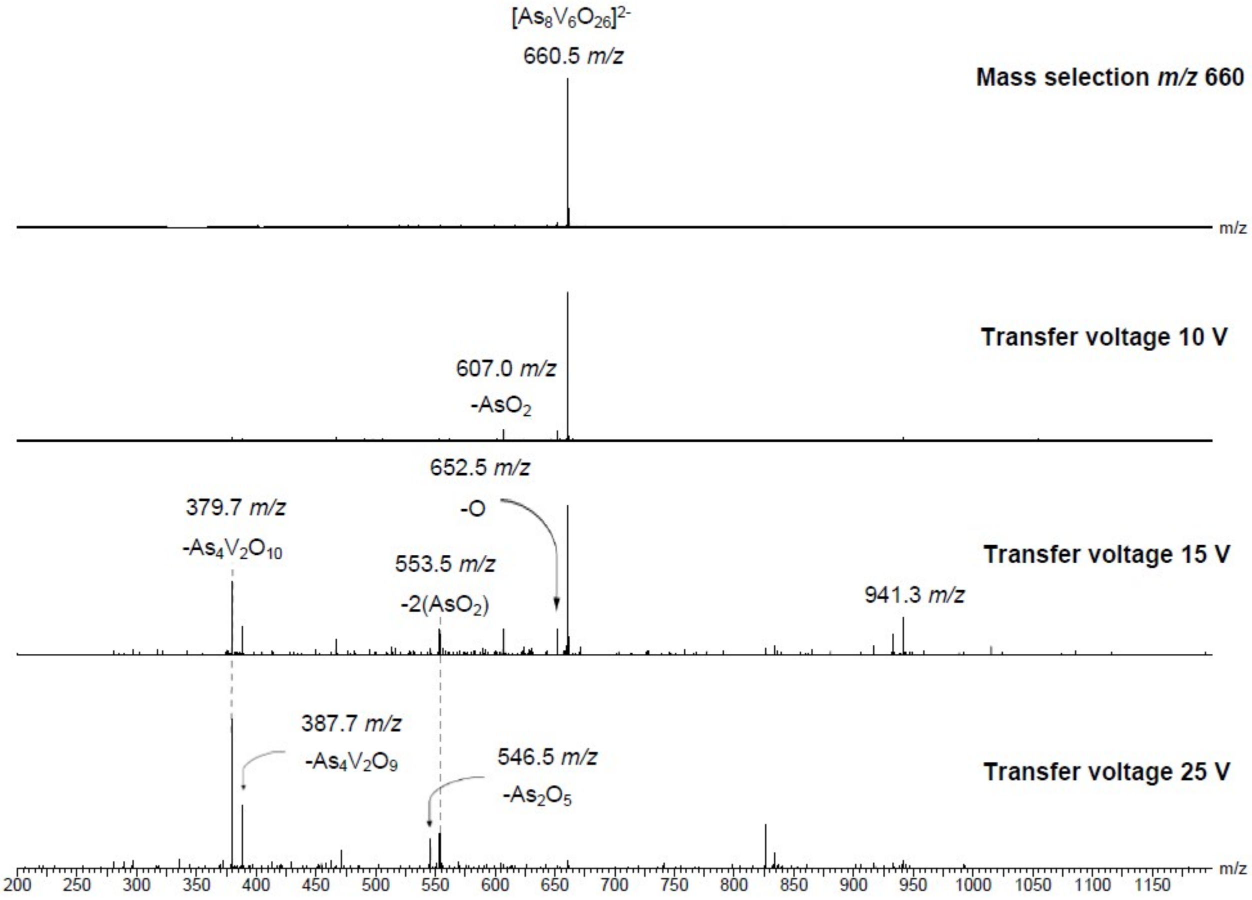

Figure S13: CID measurements of *m/z* 660 of [$V_6As_8O_{26}$]. Fragmentation proceeds via the loss of AsO_2 moieties followed by the subsequent loss of VO_8.

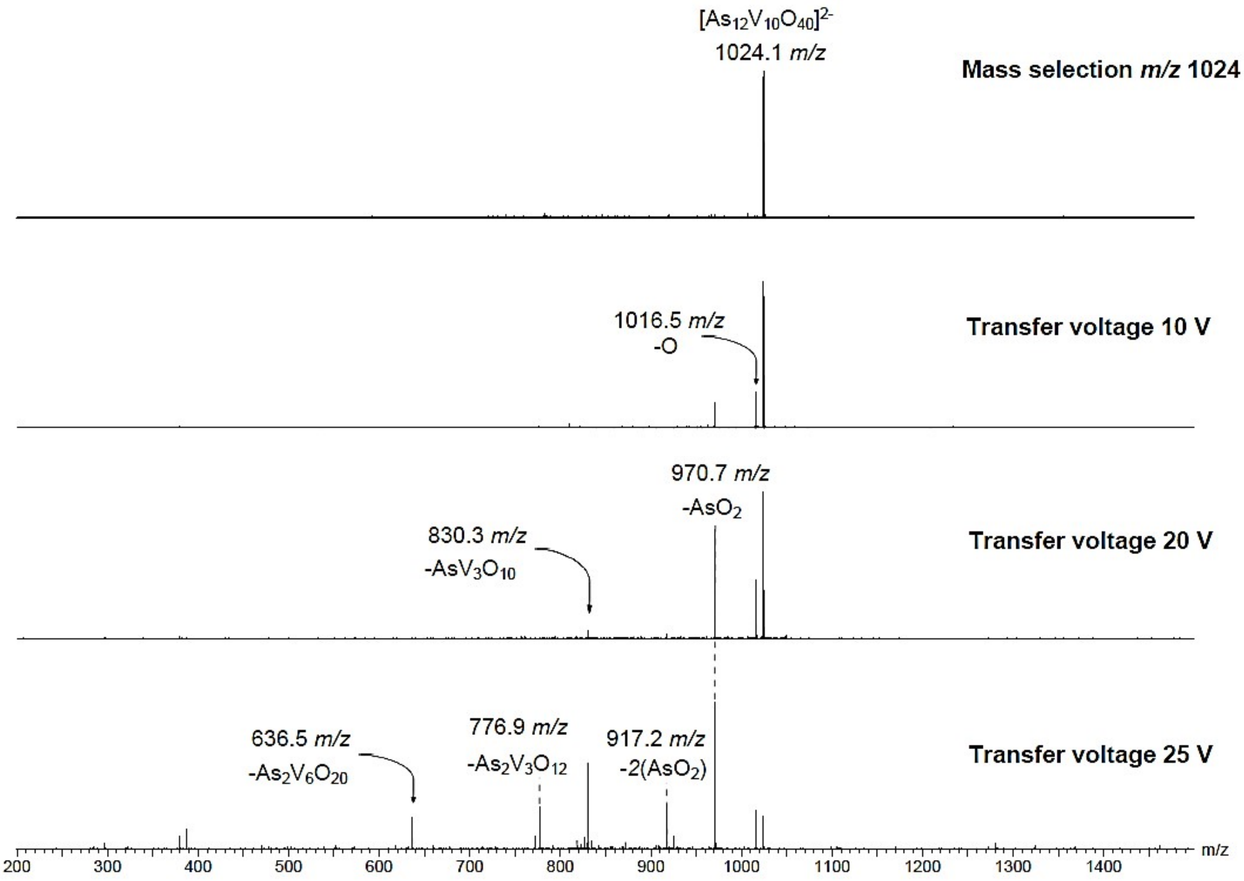

Figure S14: CID measurements of *m/z* 1024 of $[As_{12}V_{10}O_{40}]$.

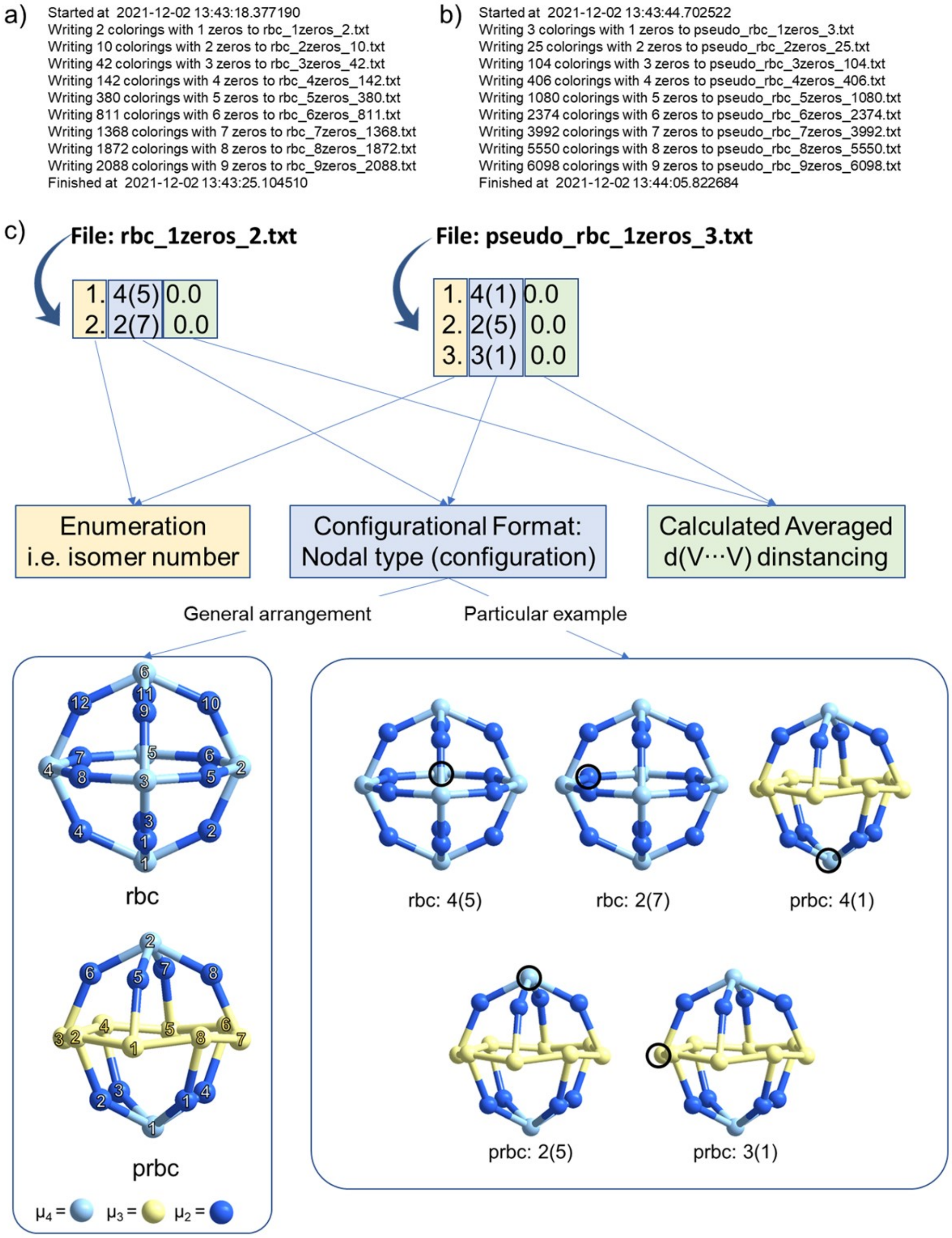

Figure S15: Program terminal message from the execution of rbc_colorings.py script (a) and pseudo_rbc_colorings.py (b). Examples of the printed isomer set files for x = 1 (c). The printed files provide information on the isomer enumeration (left), substituted configuration (middle) and averaged distance between the substituting sites (right). The substituting configuration is kept open, that is, with a consideration that all of the 18 V centres can be substituted. In this case 4, 3 and 2 inform over which metal centres does the substitution takes place. The numerical information in the parentheses that follow shows the particular position that is substituted. Degeneracies obtained through rotations are not printed in the output files.

Table S6: Calculated bond lengths in Å between V, As, terminal and bridging O atoms (i.e. O_t and O_b) for selected set of experimentally reported As-POVs and the herein proposed α-$[V_{10}As_{12}O_{40}]^{4-}$ model. All structures are optimized at COSMO/ZORA-SCALAR-UBP86/TZP level and their geometries are provided at the end of this file.

Bond lengths [Å]			
Structure	$V-O_t$	$V-O_b$	$As-O_b$
α-$[V_{10}As_{12}O_{40}]^{4-}$	1.622-1.638	1.943-2.005	1.739-1.883
β-$[V_{14}As_8O_{42}]^{4-}$	1.616-1.645	1.958-2.008	1.827-1.833
α-$[V_{14}As_8O_{42}]^{4-}$	1.617-1.633	1.954-2.025	1.819-1.829
$[V_{16}As_4O_{42}]^{8-}$	1.646-1.663	1.935-1.998	1.814-1.839
$[V_{15}As_6O_{42}]^{6-}$	1.633-1.640	1.942-2.040	1.832-1.834
$[V_6As_8O_{26}]^{4-}$	1.633-1.634	1.946-2.006	1.763-1.859

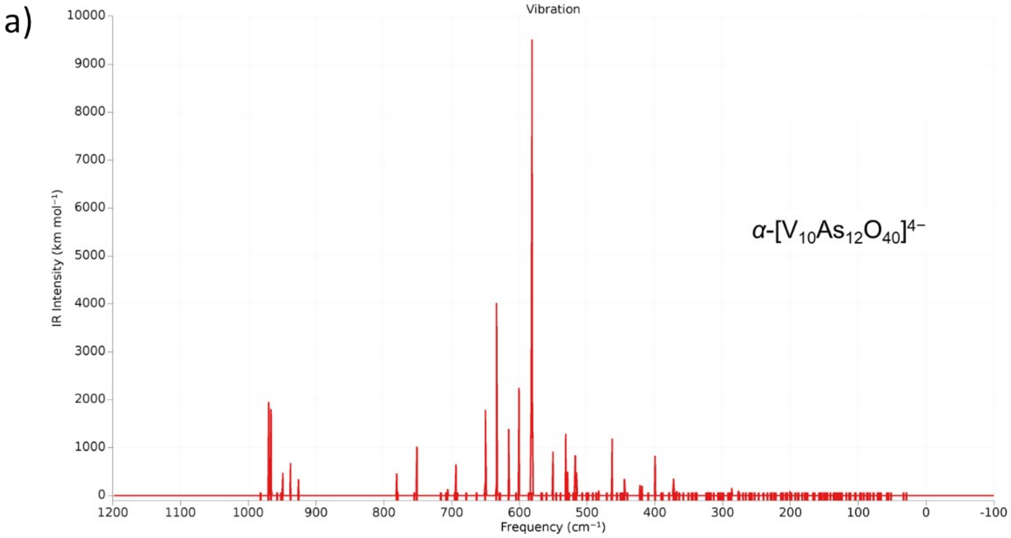

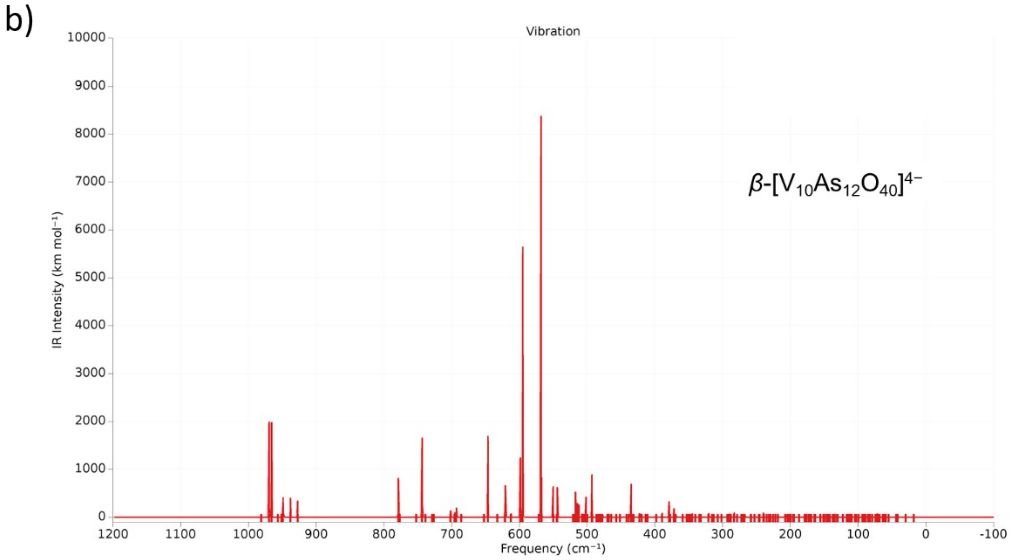

Figure S16: Frequency calculation of a) α-$[V_{10}As_{12}O_{40}]^{4-}$; b) β-$[V_{10}As_{12}O_{40}]^{4-}$ at COSMO/ZORA-SCALAR-UBP86/TZP level.

Table S7: Orbital energy values as calculated at COSMO/ZORA-SCALAR-UB3LYP/TZP level. E_{HOMO} is the energy of the α-spin HOMO, E_{LUMO} is the energy of the α-spin LUMO and $\Delta E_{HOMO-LUMO}$ is the HOMO-LUMO gap energy.

	$[V_{16}As_4O_{42}]^{8-}$	$[V_{15}As_6O_{42}]^{6-}$	α-$[V_{14}As_8O_{42}]^{4-}$	β-$[V_{14}As_8O_{42}]^{4-}$	α-$[V_{10}As_{12}O_{40}]^{4-}$	$[V_6As_8O_{26}]^{4-}$
E_{HOMO}	-5.86	-6.26	-6.70	-6.37	-6.13	-5.88
E_{LUMO}	-2.03	-2.26	-2.61	-2.75	-2.43	-1.98
$\Delta E_{HOMO-LUMO}$	3.83	4.01	4.09	3.62	3.70	3.90

Table S8: Important energy values of the isomeric $[V_{14}As_8O_{42}]^{4-}$ structures as calculated at COSMO/ZORA-SCALAR-UB3LYP/TZP level. E_{HOMO} is the energy of the α-spin HOMO, E_{LUMO} is the energy of the α-spin LUMO, $\Delta E_{HOMO-LUMO}$ is the HOMO-LUMO gap energy, E_b is the total bonding energy as obtained from the output of the single point calculations, ΔE_b is the bonding energy relative to α-$[V_{14}As_8O_{42}]^{4-}$, $d_{(V\cdots V)}$ is the interatomic distance between the substituting V atom pairs. For representation of the isomers consult figure 11.

	Isomeric $[V_{14}As_8O_{42}]^{4-}$						
	α-<br>*anti*-1,5	*β*-<br>*anti*-1,4	*γ*-<br>*syn*-1,5	*δ*-<br>*anti*-1,3	*ε*-<br>*anti*-1,2	*ζ*-<br>*anti*-1,1	*η*-<br>*syn*-1,3
E_{HOMO} [eV]	-6.13	-6.10	-6.06	-6.12	-6.00	-5.85	-4.84
E_{LUMO} [eV]	-2.43	-2.55	-2.50	-2.64	-2.68	-2.70	-2.94
$\Delta E_{HOMO-LUMO}$ [eV]	3.70	3.56	3.56	3.47	3.33	3.14	1.90
E_b [kJ·mol^{-1}]	-54386.42	-54372.77	-54368.22	-54354.63	-54342.84	-54358.39	-54342.2
ΔE_b [kJ·mol^{-1}]	0.00	13.65	18.20	31.79	43.58	28.03	44.23
$d_{(V\cdots V)}$	8.97	8.35	7.40	6.99	5.85	5.47	3.54

Cartesian coordinates (in Å)

$[V_{16}As_4O_{42}]^{8-}$
62

As	-0.00000000	-1.65898096	4.43626849
O	1.38341188	-1.44465610	3.28338305
O	0.00000000	-0.00000000	5.22866619
O	-1.38341188	-1.44465610	3.28338305
V	1.89977913	-2.80264898	1.85690358
V	2.69754338	-0.00000000	2.85918205
As	0.00000000	1.65898096	4.43626849
V	-1.89977913	-2.80264898	1.85690358
V	-2.69754338	0.00000000	2.85918205
O	2.80891594	-3.93452494	2.62438413
O	1.77992636	-3.38025691	-0.00000000
O	-0.00000000	-3.38277594	1.79696039
O	3.00223100	-1.25039188	1.41425989
O	3.00223100	1.25039188	1.41425989
O	3.94096062	-0.00000000	3.93829263
O	1.38341188	1.44465610	3.28338305
O	-1.38341188	1.44465610	3.28338305
O	-1.77992636	-3.38025691	-0.00000000
O	-2.80891594	-3.93452494	2.62438413
O	-3.00223100	-1.25039188	1.41425989
O	-3.94096062	0.00000000	3.93829263
O	-3.00223100	1.25039188	1.41425989
V	-0.00000000	-4.16423856	-0.00000000
V	1.89977913	-2.80264898	-1.85690358
V	3.59988240	-0.00000000	-0.00000000
V	1.89977913	2.80264898	1.85690358
V	-1.89977913	2.80264898	1.85690358
V	-1.89977913	-2.80264898	-1.85690358
V	-3.59988240	0.00000000	-0.00000000
O	-0.00000000	-5.82702048	-0.00000000
O	-0.00000000	-3.38277594	-1.79696039
O	2.80891594	-3.93452494	-2.62438413
O	3.00223100	-1.25039188	-1.41425989
O	1.38341188	-1.44465610	-3.28338305
O	3.00223100	1.25039188	-1.41425989
O	5.24298850	-0.00000000	-0.00000000
O	0.00000000	3.38277594	1.79696039
O	1.77992636	3.38025691	-0.00000000
O	2.80891594	3.93452494	2.62438413
O	-1.77992636	3.38025691	-0.00000000
O	-2.80891594	3.93452494	2.62438413
O	-3.00223100	-1.25039188	-1.41425989
O	-1.38341188	-1.44465610	-3.28338305
O	-2.80891594	-3.93452494	-2.62438413
O	-5.24298850	0.00000000	-0.00000000
O	-3.00223100	1.25039188	-1.41425989
V	2.69754338	-0.00000000	-2.85918205
As	-0.00000000	-1.65898096	-4.43626849
V	1.89977913	2.80264898	-1.85690358
V	0.00000000	4.16423856	-0.00000000
V	-1.89977913	2.80264898	-1.85690358
V	-2.69754338	0.00000000	-2.85918205

O	3.94096062	-0.00000000	-3.93829263
O	1.38341188	1.44465610	-3.28338305
O	0.00000000	-0.00000000	-5.22866619
O	0.00000000	3.38277594	-1.79696039
O	2.80891594	3.93452494	-2.62438413
O	0.00000000	5.82702048	-0.00000000
O	-2.80891594	3.93452494	-2.62438413
O	-1.38341188	1.44465610	-3.28338305
O	-3.94096062	0.00000000	-3.93829263
As	0.00000000	1.65898096	-4.43626849

$[V_{15}As_6O_{42}]^{6-}$
63

As	-4.50775213	-1.35874309	0.95930110
O	-5.28058525	0.00000000	0.00000000
O	-3.38681327	-2.01524476	-0.31891033
O	-3.27823460	-0.42418768	1.91344498
As	-4.50775213	1.35874309	-0.95930110
V	-2.96320450	-1.52454028	-2.21107944
V	-1.97707778	-3.42439916	0.00000000
V	-1.78706187	-1.20876118	3.06318385
V	-2.96320450	1.52454028	2.21107944
O	-3.27823460	0.42418768	-1.91344498
O	-3.38681327	2.01524476	0.31891033
O	-1.55662370	-2.81996319	-1.81030897
O	-1.47617930	-0.71357424	-3.16142678
O	-4.08131259	-2.14483964	-3.23812351
O	-2.79340354	-4.83831686	0.00000000
O	-1.66384791	-2.75805727	1.81030897
O	-0.05184652	-3.94068871	0.31891033
O	-2.46627700	-1.71638959	4.46111446
O	0.12011623	-1.63519589	3.16142678
O	-1.47617930	0.71357424	3.16142678
O	-4.08131259	2.14483964	3.23812351
O	-1.55662370	2.81996319	1.81030897
V	-1.78706187	1.20876118	-3.06318385
V	-1.97707778	3.42439916	0.00000000
V	-0.15328696	-2.15202157	-3.06318385
V	0.16131164	-3.32848051	2.21107944
As	1.07717003	-4.58319941	-0.95930110
V	1.94034882	-0.94326038	3.06318385
V	-0.15328696	2.15202157	3.06318385
O	0.12011623	1.63519589	-3.16142678
O	-2.46627700	1.71638959	-4.46111446
O	-1.66384791	2.75805727	-1.81030897
O	-2.79340354	4.83831686	0.00000000
O	-0.05184652	3.94068871	-0.31891033
O	-0.25329849	-2.99405333	-4.46111446
O	1.35606307	-0.92162165	-3.16142678
O	1.27176000	-3.05112828	-1.91344498
O	0.18317068	-4.60694021	3.23812351
O	2.00647461	-2.62694061	1.91344498
O	2.64029262	-4.57312097	0.00000000
O	2.71957549	-1.27766373	4.46111446
O	1.35606307	0.92162165	3.16142678
O	3.22047161	-0.06190593	1.81030897
O	-0.25329849	2.99405333	4.46111446

O	1.27176000	3.05112828	1.91344498
V	0.16131164	3.32848051	-2.21107944
V	1.94034882	0.94326038	-3.06318385
As	1.07717003	4.58319941	0.95930110
V	2.80189286	-1.80394024	-2.21107944
As	3.43058210	-3.22445632	0.95930110
V	2.80189286	1.80394024	2.21107944
V	3.95415555	-0.00000000	0.00000000
O	2.00647461	2.62694061	-1.91344498
O	0.18317068	4.60694021	-3.23812351
O	3.22047161	0.06190593	-1.81030897
O	2.71957549	1.27766373	-4.46111446
O	2.64029262	4.57312097	0.00000000
O	3.89814191	-2.46210057	-3.23812351
O	3.43865979	-1.92544395	-0.31891033
O	3.89814191	2.46210057	3.23812351
O	3.43865979	1.92544395	0.31891033
O	5.58680708	-0.00000000	0.00000000
As	3.43058210	3.22445632	-0.95930110

α-$[V_{14}As_8O_{42}]^{4-}$
64

As	0.97429799	-3.32456173	3.20121156
V	-0.00000000	0.00000000	3.80552380
V	2.04775425	-2.04775425	-2.49525411
V	-0.00000000	-3.96786925	-0.00000000
V	2.87864494	-2.87864494	0.26429561
O	1.49194893	-3.26631242	-1.05679583
O	1.33130042	-3.41066070	1.42014207
O	-0.00000000	-5.59370713	-0.00000000
O	-0.14454436	-1.87157475	3.13365128
O	-4.03326818	-4.03326818	-0.28174031
O	4.03326818	-4.03326818	0.28174031
O	2.49716609	-2.49716609	3.78559669
O	2.89463981	-2.89463981	-3.59257186
O	-0.00000000	0.00000000	5.42255403
O	3.26631242	-1.49194893	-1.05679583
V	3.96786925	-0.00000000	-0.00000000
O	3.26631242	1.49194893	1.05679583
V	2.04775425	2.04775425	2.49525411
O	1.49194893	3.26631242	1.05679583
V	0.00000000	3.96786925	-0.00000000
O	-1.49194893	3.26631242	-1.05679583
V	-2.04775425	2.04775425	-2.49525411
O	-3.26631242	1.49194893	-1.05679583
V	-3.96786925	0.00000000	-0.00000000
O	-3.26631242	-1.49194893	1.05679583
V	-2.04775425	-2.04775425	2.49525411
O	-1.49194893	-3.26631242	1.05679583
O	-1.33130042	-3.41066070	-1.42014207
As	-0.97429799	-3.32456173	-3.20121156
O	0.14454436	-1.87157475	-3.13365128
V	-0.00000000	0.00000000	-3.80552380
O	-1.87157475	0.14454436	-3.13365128
As	-3.32456173	-0.97429799	-3.20121156

O	-3.41066070	-1.33130042	-1.42014207
O	-3.41066070	1.33130042	1.42014207
As	-3.32456173	0.97429799	3.20121156
O	-1.87157475	-0.14454436	3.13365128
As	3.32456173	-0.97429799	3.20121156
O	3.41066070	-1.33130042	1.42014207
O	3.41066070	1.33130042	-1.42014207
As	3.32456173	0.97429799	-3.20121156
O	1.87157475	-0.14454436	-3.13365128
O	-0.14454436	1.87157475	-3.13365128
As	0.97429799	3.32456173	-3.20121156
O	1.33130042	3.41066070	-1.42014207
O	-1.33130042	3.41066070	1.42014207
As	-0.97429799	3.32456173	3.20121156
O	0.14454436	1.87157475	3.13365128
O	1.87157475	0.14454436	3.13365128
O	5.59370713	-0.00000000	-0.00000000
O	2.89463981	2.89463981	3.59257186
O	-2.49716609	-2.49716609	-3.78559669
O	-2.49716609	2.49716609	3.78559669
O	-2.89463981	-2.89463981	3.59257186
V	-2.87864494	2.87864494	0.26429561
O	-4.03326818	4.03326818	0.28174031
O	0.00000000	5.59370713	-0.00000000
V	2.87864494	2.87864494	-0.26429561
O	4.03326818	4.03326818	-0.28174031
O	-5.59370713	0.00000000	-0.00000000
O	2.49716609	2.49716609	-3.78559669
V	-2.87864494	-2.87864494	-0.26429561
O	-2.89463981	2.89463981	-3.59257186
O	-0.00000000	0.00000000	-5.42255403

β-$[V_{14}As_8O_{42}]^{4-}$
64

As	4.28231729	0.00000000	-1.64496253
O	1.37700559	3.09148680	1.42886997
As	-4.28231729	0.00000000	1.64496253
O	-3.09148680	-1.37700559	-1.42886997
As	4.28231729	0.00000000	1.64496253
O	-3.09148680	1.37700559	-1.42886997
As	-4.28231729	0.00000000	-1.64496253
O	2.72336556	-2.72336556	-4.04150907
As	0.00000000	-4.28231729	-1.64496253
O	-1.37700559	3.09148680	1.42886997
As	0.00000000	4.28231729	1.64496253
O	-3.09148680	1.37700559	1.42886997
As	0.00000000	-4.28231729	1.64496253
O	1.79165124	0.00000000	3.51947228
As	0.00000000	4.28231729	-1.64496253
O	0.00000000	-1.79165124	3.51947228
V	1.87446231	1.87446231	-2.94687360
O	0.00000000	1.79165124	3.51947228
V	-1.87446231	-1.87446231	2.94687360
O	0.00000000	-5.07656412	0.00000000
V	2.69740044	2.69740044	0.00000000
O	-1.37700559	-3.09148680	-1.42886997

V	-2.69740044	-2.69740044	0.00000000
O	3.84029786	-3.84029786	0.00000000
V	1.87446231	1.87446231	2.94687360
O	0.00000000	1.79165124	-3.51947228
V	-1.87446231	-1.87446231	-2.94687360
O	-2.72336556	2.72336556	-4.04150907
V	-1.87446231	1.87446231	2.94687360
O	3.09148680	-1.37700559	-1.42886997
V	1.87446231	-1.87446231	-2.94687360
O	2.72336556	2.72336556	4.04150907
V	0.00000000	0.00000000	4.30879886
O	-1.79165124	0.00000000	-3.51947228
V	0.00000000	0.00000000	-4.30879886
O	0.00000000	0.00000000	5.95404641
V	1.87446231	-1.87446231	2.94687360
O	0.00000000	0.00000000	-5.95404641
V	-1.87446231	1.87446231	-2.94687360
O	-1.79165124	0.00000000	3.51947228
V	2.69740044	-2.69740044	0.00000000
O	3.09148680	-1.37700559	1.42886997
V	-2.69740044	2.69740044	0.00000000
O	1.37700559	-3.09148680	-1.42886997
O	5.07656412	0.00000000	0.00000000
O	1.37700559	-3.09148680	1.42886997
O	-5.07656412	0.00000000	0.00000000
O	1.79165124	0.00000000	-3.51947228
O	3.09148680	1.37700559	-1.42886997
O	0.00000000	-1.79165124	-3.51947228
O	-3.09148680	-1.37700559	1.42886997
O	0.00000000	5.07656412	0.00000000
O	2.72336556	2.72336556	-4.04150907
O	-2.72336556	-2.72336556	-4.04150907
O	-2.72336556	-2.72336556	4.04150907
O	-2.72336556	2.72336556	4.04150907
O	1.37700559	3.09148680	-1.42886997
O	2.72336556	-2.72336556	4.04150907
O	-1.37700559	-3.09148680	1.42886997
O	-3.84029786	3.84029786	0.00000000
O	3.84029786	3.84029786	0.00000000
O	-1.37700559	3.09148680	-1.42886997
O	-3.84029786	-3.84029786	0.00000000
O	3.09148680	1.37700559	1.42886997

$[V_6As_8O_{26}]^{4-}$
40

As	3.33943286	-1.01407099	1.70622261
O	1.85976182	0.14387383	1.67633939
O	3.47489091	-1.52951617	0.02570091
O	2.43732153	-2.43732153	2.49193280
V	0.00000000	0.00000000	2.33648244
V	2.08328934	2.08328934	1.21712092
V	2.08328934	-2.08328934	-1.21712092
As	1.01407099	-3.33943286	1.70622261
O	0.00000000	0.00000000	3.96924940
O	-1.85976182	-0.14387383	1.67633939
O	-0.14387383	-1.85976182	1.67633939

O	0.14387383	1.85976182	1.67633939
O	2.84935759	2.84935759	2.44095288
O	3.47489091	1.52951617	-0.02570091
O	1.52951617	3.47489091	-0.02570091
O	1.85976182	-0.14387383	-1.67633939
O	0.14387383	-1.85976182	-1.67633939
O	2.84935759	-2.84935759	-2.44095288
O	1.52951617	-3.47489091	0.02570091
As	-3.33943286	1.01407099	1.70622261
V	-2.08328934	-2.08328934	1.21712092
As	-1.01407099	3.33943286	1.70622261
As	3.33943286	1.01407099	-1.70622261
As	1.01407099	3.33943286	-1.70622261
V	0.00000000	0.00000000	-2.33648244
As	-1.01407099	-3.33943286	-1.70622261
O	-3.47489091	1.52951617	0.02570091
O	-2.43732153	2.43732153	2.49193280
O	-2.84935759	-2.84935759	2.44095288
O	-3.47489091	-1.52951617	-0.02570091
O	-1.52951617	-3.47489091	-0.02570091
O	-1.52951617	3.47489091	0.02570091
O	2.43732153	2.43732153	-2.49193280
O	-0.14387383	1.85976182	-1.67633939
O	0.00000000	0.00000000	-3.96924940
O	-1.85976182	0.14387383	-1.67633939
O	-2.43732153	-2.43732153	-2.49193280
V	-2.08328934	2.08328934	-1.21712092
As	-3.33943286	-1.01407099	-1.70622261
O	-2.84935759	2.84935759	-2.44095288

ζ-$[V_{10}As_{12}O_{40}]^{4-}$ (i.e. *anti*-1,1)
62

O	-0.00000000	-3.64796208	3.69889010
As	1.66549975	2.98758673	4.04091703
As	-2.97654079	3.06227310	-1.87585799
O	2.29551203	3.64662697	2.47067776
O	-1.32305146	3.47493875	-2.21696495
As	3.65820716	3.02023639	1.40334879
V	0.00000000	2.73627424	-3.47707395
As	2.97654079	3.06227310	-1.87585799
O	-2.29551203	3.64662697	2.47067776
O	2.86432660	3.64244613	-0.07685563
O	1.32305146	3.47493875	-2.21696495
O	0.00000000	3.72803291	-4.78489634
O	-3.13999461	1.23342094	1.29369784
O	-2.84376391	1.22312858	-1.56573266
O	0.00000000	3.64796208	3.69889010
O	-1.26574691	1.26138973	-3.90473607
O	1.26574691	1.26138973	-3.90473607
As	-1.66549975	2.98758673	4.04091703
O	2.84376391	1.22312858	-1.56573266
O	3.13999461	1.23342094	1.29369784
As	-3.65820716	3.02023639	1.40334879
O	-1.41548869	1.22475338	3.55071146
O	1.41548869	1.22475338	3.55071146
V	-3.62259099	0.00000000	-0.22417226

V	-2.82052290	0.00000000	2.81289317
V	-2.54081746	0.00000000	-3.14413113
V	-0.00000000	-0.00000000	-4.68824548
V	2.54081746	-0.00000000	-3.14413113
V	3.62259099	-0.00000000	-0.22417226
V	2.82052290	-0.00000000	2.81289317
V	-0.00000000	-0.00000000	4.23063112
O	-5.23908850	0.00000000	-0.37221074
O	-3.13999461	-1.23342094	1.29369784
O	-2.84376391	-1.22312858	-1.56573266
O	-4.10395041	0.00000000	3.79866395
O	-1.41548869	-1.22475338	3.55071146
O	-3.89497927	0.00000000	-4.06659667
O	-1.26574691	-1.26138973	-3.90473607
O	-0.00000000	-0.00000000	-6.34971343
O	1.26574691	-1.26138973	-3.90473607
O	3.89497927	-0.00000000	-4.06659667
O	2.84376391	-1.22312858	-1.56573266
O	5.23908850	-0.00000000	-0.37221074
O	3.13999461	-1.23342094	1.29369784
O	4.10395041	-0.00000000	3.79866395
O	1.41548869	-1.22475338	3.55071146
O	-0.00000000	-0.00000000	5.84777314
O	2.29551203	-3.64662697	2.47067776
O	2.86432660	-3.64244613	-0.07685563
As	-2.97654079	-3.06227310	-1.87585799
As	-1.66549975	-2.98758673	4.04091703
As	2.97654079	-3.06227310	-1.87585799
O	-2.29551203	-3.64662697	2.47067776
O	1.32305146	-3.47493875	-2.21696495
As	-3.65820716	-3.02023639	1.40334879
V	-0.00000000	-2.73627424	-3.47707395
O	-2.86432660	3.64244613	-0.07685563
O	-2.86432660	-3.64244613	-0.07685563
O	-1.32305146	-3.47493875	-2.21696495
O	-0.00000000	-3.72803291	-4.78489634
As	1.66549975	-2.98758673	4.04091703
As	3.65820716	-3.02023639	1.40334879

ε-$[V_{10}As_{12}O_{40}]^{4-}$ (i.e. *anti*-1,2)
62

O	0.16160412	-3.37051482	4.68210857
As	-1.78654754	3.09451827	3.98417369
As	-0.55901988	3.35246200	-3.08380643
O	-0.67645734	3.39802932	2.56224683
O	0.95727133	3.34452531	-2.21209750
As	1.11298530	3.19392862	2.94839124
V	2.67189578	2.55287067	-2.60212163
As	3.39350804	2.96059529	0.78883771
O	-3.19253536	3.55501681	0.50598212
O	1.58660078	3.37632597	1.24040372
O	3.33684927	3.37503170	-0.87562385
O	3.58658692	3.44711354	-3.63164335
O	-3.50834443	1.09187283	1.69463050
O	-2.86357860	1.28383699	-1.08814260
O	-3.40142623	3.42338255	3.22239994

O	-0.96127331	1.54898616	-3.26538638
O	1.85622072	1.24580868	-3.86671433
As	-4.26198570	2.76490160	1.74935448
O	3.49476048	0.86857679	-2.02915623
O	3.02577062	1.11512079	0.77088433
As	-3.24543686	3.08190022	-1.27526116
O	-1.69573649	1.25316005	3.87521090
O	1.13360131	1.31260148	3.05716108
V	-3.60666789	-0.12243802	0.08790086
V	-2.95665162	-0.10985959	3.18830121
V	-2.33776668	0.20064254	-2.73979476
V	0.20442036	0.38847473	-4.43078427
V	2.94133967	-0.32945476	-3.48509511
V	3.70119868	-0.27349775	-0.49369505
V	2.57665750	0.07573752	2.41404135
V	-0.00793917	0.19201775	4.24925644
O	-5.18529003	-0.34935299	-0.20208464
O	-3.02970176	-1.30648286	1.60041732
O	-2.61273015	-1.14182831	-1.29845875
O	-4.23371370	-0.33272394	4.15737225
O	-1.44147147	-1.15716648	3.99253348
O	-3.64922206	0.29687899	-3.70726677
O	-1.05871458	-0.89528902	-3.66420116
O	-0.10206700	0.70431124	-6.02202155
O	1.34478872	-1.17183551	-4.22397819
O	4.19363471	-0.54763283	-4.53723489
O	3.06295431	-1.56147448	-1.91177116
O	5.29724605	-0.49679550	-0.24620922
O	2.75714809	-1.29305960	1.00327350
O	3.90374758	0.19680279	3.33757038
O	1.33270432	-1.07475416	3.53095359
O	0.34017895	0.44748025	5.80867261
O	1.45270677	-3.43940433	2.22450440
O	1.89911653	-3.51735514	-0.37082639
As	-2.55647161	-2.99934942	-1.62438962
As	-1.58610439	-2.94478153	4.40336179
As	2.88774042	-3.42437146	-1.98946565
O	-1.71757558	-3.55101184	2.68637577
O	1.58469394	-3.65001088	-3.09960608
As	-3.12934572	-3.17409395	1.57385689
V	0.03413957	-2.54722423	-3.71902938
O	-1.57280908	3.68296392	-1.53604117
O	-2.11633346	-3.48155092	0.13117227
O	-0.93264104	-3.24545796	-2.17938326
O	-0.44011731	-3.34836842	-5.06957035
As	1.72522779	-2.82368839	3.92353177
As	2.86837739	-3.14504632	1.08597984

δ-$[V_{10}As_{12}O_{40}]^{4-}$ (i.e. *anti*-1,3)
62

O	0.14439799	-3.31148394	4.75527636
As	-3.73539256	2.95089873	1.59545100
As	1.26619465	3.23880898	-2.40195338
O	-2.09988765	3.64546449	2.00727211
O	1.73234165	3.04206861	-0.73010682
As	-1.07885545	3.18366142	3.45298995

V	3.37202802	2.33178363	0.01449790
As	2.16048702	3.05304362	3.20234105
O	-3.26348162	3.72048649	-2.77359243
O	0.44213370	3.54157845	2.57282916
O	2.99885556	3.33078155	1.72840283
O	4.69609374	3.17423729	-0.45542539
O	-3.20475153	1.20416738	1.31768660
O	-3.40495897	1.20814899	-1.51425232
O	-3.57947624	3.58775051	-0.09988747
O	-1.73947110	1.56974064	-3.78013464
O	1.23804227	1.48737195	-3.09691927
As	-4.29799572	2.80430317	-1.59546417
O	3.34666240	0.85242657	-1.30254657
O	3.77862410	0.75035957	1.12906869
As	-1.62435451	3.37410349	-3.52355821
O	-1.07063033	1.33710541	3.23018137
O	1.82561112	1.18568620	3.13682348
V	-3.71313448	-0.14171435	-0.06880053
V	-2.56540846	0.09890838	2.86633078
V	-2.84643990	0.05331344	-3.09174925
V	-0.06034293	0.52637390	-4.27957399
V	2.60986774	0.02490797	-2.89509118
V	4.00168853	-0.61047756	-0.24856691
V	3.17625161	-0.19988554	2.69621202
V	0.36744462	0.22152116	4.08541694
O	-5.30864916	-0.41378665	-0.01933981
O	-2.90527577	-1.22490302	1.43408600
O	-2.90370628	-1.19976018	-1.53856252
O	-3.76275180	0.20837353	3.95176280
O	-1.15717947	-1.02877123	3.76257101
O	-4.15540704	-0.16329428	-4.03798934
O	-1.35188801	-0.86294144	-3.87940580
O	0.17304048	0.82702194	-5.87756920
O	1.11694730	-0.90760686	-3.73716181
O	3.75640635	0.16567647	-4.05911777
O	2.99104114	-1.55232517	-1.71586151
O	5.60048436	-0.90953211	-0.47757699
O	3.30866016	-1.66905877	1.33440072
O	4.42469078	-0.19655436	3.74211908
O	1.68867134	-1.19488080	3.69544852
O	0.42681749	0.63045293	5.65271441
O	1.59308690	-3.61115244	2.44642276
O	1.89829602	-3.52128157	-0.15084834
As	-2.85074632	-3.09049285	-1.68383507
As	-1.51046869	-2.75626004	4.24748010
As	2.68974964	-3.38338295	-1.86099241
O	-1.50353886	-3.41577256	2.53303638
O	1.21777887	-3.51214079	-2.76822527
As	-2.92679680	-3.10396111	1.43286926
V	-0.11279830	-2.42805753	-3.73039415
O	-0.57349808	3.32502178	-2.03939945
O	-1.93871966	-3.31715612	-0.05314560
O	-1.49767726	-3.38717014	-2.70936018
O	-0.12659631	-3.08109296	-5.23302814
As	1.82799445	-2.96592010	4.13421905
As	2.99988925	-3.49473118	1.27152552

β-$[V_{10}As_{12}O_{40}]^{4-}$ (i.e. *anti*-1,4)
62

V	1.06438846	-3.12739288	-3.00354663
O	2.55341122	-2.97734730	-1.78762094
O	1.54531392	-1.27039363	-3.28753495
O	0.57640481	-4.41322909	-1.51237603
O	-0.71188711	-2.22130232	-3.08181069
V	-1.66374992	2.95950851	2.66338504
V	1.97017527	-3.85906048	-0.17405888
O	-2.37775191	3.69805953	3.92664135
V	-0.14303078	-0.36296133	-3.58866165
O	0.10508327	2.21193305	3.23927720
As	-0.97896191	-5.38465134	-1.46890352
As	-2.38202502	-3.00851904	-3.02730723
O	2.52260079	-2.30983956	0.94626406
O	0.55000792	-3.70468563	1.23596336
As	-3.73013281	0.27834717	2.80055108
O	1.37794068	-4.01002973	-4.34897436
O	-3.24264443	-1.40439784	2.11164362
O	0.53606557	1.32759046	-2.81761780
O	-1.74762283	0.50296416	-2.72244708
O	2.94303072	-5.12097724	0.15669178
O	-2.80137102	3.09796131	1.11305919
O	-1.08467574	-5.81572179	0.31472137
O	-2.15101896	-3.97482423	-1.46727791
O	-3.28554027	-1.72070489	-2.14317814
V	1.22706437	-2.26760141	2.44988096
O	-0.70442080	4.29031329	1.51507504
As	-0.67212004	-4.92018187	1.85098545
V	-1.15919782	2.37883224	-2.59937638
O	-4.41160962	0.85776526	1.31444434
As	-3.54776530	0.00020186	-2.86369705
O	-0.60213148	-1.59896467	2.83894266
O	1.46698145	-0.31269423	2.81601599
O	-0.37085161	0.10663695	5.14418191
O	-5.29231147	3.04952019	-0.31307657
O	-2.11032127	-3.76534686	1.85036797
O	-2.57763118	2.82289612	-1.34954638
O	-0.26990958	3.68994109	-1.35351796
O	1.84185691	-3.03108529	3.74107822
O	-2.44125138	5.62527852	-0.47906962
O	-4.26311916	0.63009185	-1.40371926
V	-0.28913944	0.26285972	3.53223304
As	-2.23100861	-2.47812230	3.14802925
O	-1.47293290	3.13761622	-4.00718933
V	-1.80633330	4.14668336	-0.16430091
V	-3.97252356	2.09799567	-0.14462388
O	-0.32848479	-0.19396137	-5.19972788
O	-1.96751043	0.99669835	2.78754709
As	1.63965060	2.99448404	3.88601851
As	2.31804965	1.83910384	-3.10248451
O	2.77595467	2.17002917	2.69062459
O	3.19456404	0.64974498	-2.15264066
As	3.18690999	0.41377646	3.02609079
V	3.33380994	-1.25067986	-2.41045757
As	4.29393225	-1.62320800	0.87243449

O	1.80160281	4.44729926	0.36639594
O	3.76793564	0.13660438	1.34770869
O	4.52032982	-1.33675188	-0.81564108
O	4.38872413	-1.55987570	-3.62233548
O	1.59923785	4.64215356	3.11416126
As	0.79121336	5.32673030	1.60955563
As	1.37599055	4.51670404	-1.43043301
O	2.45183320	3.10659884	-1.74538188

α-$[V_{10}As_{12}O_{40}]^{4-}$ (i.e. *anti*-1,5)
62

V	1.29047794	-2.76629225	-2.53708467
O	2.67424619	-3.08089665	-1.24117264
O	1.69937035	-0.81608534	-2.57713254
O	0.53791802	-4.31218148	-1.44917363
O	-0.49336274	-1.85531113	-2.63608353
V	-1.29047794	2.76629225	2.53708467
V	1.91280703	-4.36672457	-0.00000000
O	-1.70759163	3.37285759	3.98933847
V	0.00000000	0.00000000	-3.20995337
As	3.47384198	-0.22776377	-2.81301758
As	-0.99973026	-5.28486495	-1.68900483
As	-2.11640077	-2.66927615	-3.04336655
O	2.67424619	-3.08089665	1.24117264
O	0.53791802	-4.31218148	1.44917363
As	-3.47384198	0.22776377	2.81301758
O	5.29606958	-3.05431659	-0.00000000
O	4.24185401	-0.77605394	-1.35249918
O	0.49336274	1.85531113	-2.63608353
O	-1.69937035	0.81608534	-2.57713254
As	2.11640077	2.66927615	3.04336655
O	3.18972746	1.51729947	-2.16382633
O	-1.29858964	-5.92059398	-0.00000000
O	-2.16762834	-3.86497065	-1.64701917
O	-3.18972746	-1.51729947	-2.16382633
V	1.29047794	-2.76629225	2.53708467
V	3.93183793	-2.15418058	-0.00000000
As	-0.99973026	-5.28486495	1.68900483
V	-1.29047794	2.76629225	-2.53708467
As	2.11640077	2.66927615	-3.04336655
As	-3.47384198	0.22776377	-2.81301758
O	-0.49336274	-1.85531113	2.63608353
O	1.69937035	-0.81608534	2.57713254
O	0.00000000	0.00000000	4.83225705
O	4.24185401	-0.77605394	1.35249918
O	-2.16762834	-3.86497065	1.64701917
O	-2.67424619	3.08089665	-1.24117264
O	-0.53791802	4.31218148	-1.44917363
As	0.99973026	5.28486495	1.68900483
O	2.16762834	3.86497065	-1.64701917
O	-4.24185401	0.77605394	-1.35249918
V	0.00000000	0.00000000	3.20995337
As	-2.11640077	-2.66927615	3.04336655
As	3.47384198	-0.22776377	2.81301758
V	-1.91280703	4.36672457	-0.00000000
V	-3.93183793	2.15418058	-0.00000000
As	0.99973026	5.28486495	-1.68900483

O	-1.69937035	0.81608534	2.57713254
O	0.49336274	1.85531113	2.63608353
O	2.16762834	3.86497065	1.64701917
O	-3.18972746	-1.51729947	2.16382633
O	3.18972746	1.51729947	2.16382633
O	-2.67424619	3.08089665	1.24117264
O	-0.53791802	4.31218148	1.44917363
O	-4.24185401	0.77605394	1.35249918
O	-5.29606958	3.05431659	-0.00000000
O	1.29858964	5.92059398	-0.00000000
O	-2.73407567	5.78395681	-0.00000000
O	-1.70759163	3.37285759	-3.98933847
O	0.00000000	0.00000000	-4.83225705
O	1.70759163	-3.37285759	-3.98933847
O	2.73407567	-5.78395681	-0.00000000
O	1.70759163	-3.37285759	3.98933847

η-$[V_{10}As_{12}O_{40}]^{4-}$ (i.e. *syn*-1,3)
62

V	-2.68399240	2.70695621	0.19187233
O	-1.31419435	3.07613743	-1.10432181
O	-3.31275958	1.17661330	1.37162650
O	-1.12363217	3.30248932	1.34247582
O	-3.07928646	1.31589401	-1.09607646
O	-3.83831089	3.87470925	0.19371666
V	0.11476939	3.96000257	-0.14321190
O	2.75854908	-2.63402162	-3.64495793
V	-4.02436821	-0.02711827	-0.07897810
As	-3.30633955	1.11686655	3.20235104
As	-1.11122837	3.31729209	3.17098228
O	3.82449272	-3.78358123	0.26230950
O	1.48997908	3.25569618	-1.29409660
O	1.75924758	3.82261182	1.09822215
O	-0.08388439	5.56665576	-0.39010264
V	1.81348372	1.88108227	-2.69870886
As	-1.55900543	-3.86694701	-3.24303123
O	-3.76688429	-1.71175557	1.03222386
O	-3.49791175	-1.44122396	-1.36509115
O	-5.62908578	0.23652207	-0.21664400
O	-2.96785713	-0.66862926	3.41682547
O	-1.53986184	1.54784507	3.42476942
O	0.66035656	2.98625793	3.45288523
V	-1.72632174	1.80326874	-2.56037166
V	3.08432912	3.10875011	-0.21675782
O	1.52078299	-1.53352711	3.33797922
As	1.85277761	4.25943569	2.84814163
V	-3.13278023	-3.05698662	-0.30229641
As	-4.24173696	-1.83515207	2.78407303
O	0.02532507	1.89723396	-3.14488627
O	3.27999257	1.49570047	-1.29719012
O	3.84527982	1.82068369	1.11212373
O	4.19645032	4.24903805	-0.61517198
O	-2.72221611	-2.61687347	-3.81131352
O	3.37852833	3.40528475	3.38384795
O	-1.79352801	-3.76681178	0.99237787
O	-1.54680111	-3.51833249	-1.42716966

O	-4.29709063	-4.16301253	-0.56092124
O	-3.41246729	-3.38617592	3.27319603
O	0.02010325	-3.00473530	-3.63050496
V	4.05553940	0.19955969	-0.10699155
As	1.55990639	-3.82899469	-3.03104107
As	4.23909375	1.88427281	2.87353172
V	-0.13048012	-4.06121092	-0.13838974
As	-1.88617487	-4.22749779	2.75219310
O	1.81042031	-0.04921381	-3.12848358
O	3.37950634	-1.30457814	-1.22652547
O	3.21757999	-1.06772800	1.21503922
O	5.68016936	0.06832665	-0.22190138
O	-1.71443623	-0.11157285	-3.05835971
O	2.94039709	0.66564266	3.39339237
O	1.08191380	-3.15337585	1.16282895
O	1.36519060	-3.32103729	-1.27054116
O	0.08860401	-5.67989938	-0.14783396
O	-0.68232168	-2.94628180	3.32354789
As	3.30426324	-0.93767980	-3.06913382
V	2.69165755	-2.63714078	0.04227009
As	-3.24128007	-0.93831622	-3.15241397
As	3.27845433	-1.08748304	3.06352597
As	1.07984660	-3.27626937	3.01534499
O	2.61191802	2.64435943	-3.91583924
O	-2.62449607	2.60462814	-3.68065833

γ-$[V_{10}As_{12}O_{40}]^{4-}$ (i.e. *syn*-1,5)

62

V	-2.62202146	3.01846721	-0.16995966
O	-1.23910945	3.89472589	-1.17713609
O	-2.92649650	1.41785111	1.01177751
O	-1.46762908	3.83828409	1.27927716
O	-2.69161959	1.48487456	-1.41472021
O	-4.01191491	3.86180112	-0.26246849
V	0.00000000	4.59002779	0.13560102
V	0.00000000	3.70064783	-2.72403946
V	-3.41604963	-0.00000000	-0.32132411
As	-3.73089395	1.59624308	2.65448208
As	-1.64440388	4.08340158	3.08229447
As	-2.67735125	1.57341061	-3.32916148
O	1.23910945	3.89472589	-1.17713609
O	1.46762908	3.83828409	1.27927716
O	0.00000000	6.22596304	0.20805696
O	1.37256665	-2.67348336	-3.66068952
O	-1.37256665	2.67348336	-3.66068952
O	-2.92649650	-1.41785111	1.01177751
O	-2.69161959	-1.48487456	-1.41472021
O	-5.03308490	-0.00000000	-0.45709594
O	-3.18813738	-0.00000000	3.38169537
O	-2.40247302	2.47276789	3.55323810
O	0.00000000	3.49004118	3.63981276
O	-1.72809201	0.00000000	-3.53057017
V	2.62202146	3.01846721	-0.16995966
O	2.40247302	-2.47276789	3.55323810
As	1.64440388	4.08340158	3.08229447
V	-2.62202146	-3.01846721	-0.16995966
As	-3.73089395	-1.59624308	2.65448208
As	-2.67735125	-1.57341061	-3.32916148

O	2.69161959	1.48487456	-1.41472021
O	2.92649650	1.41785111	1.01177751
O	4.01191491	3.86180112	-0.26246849
O	1.37256665	2.67348336	-3.66068952
O	2.40247302	2.47276789	3.55323810
O	-1.46762908	-3.83828409	1.27927716
O	-1.23910945	-3.89472589	-1.17713609
O	-4.01191491	-3.86180112	-0.26246849
O	-2.40247302	-2.47276789	3.55323810
O	-1.37256665	-2.67348336	-3.66068952
V	3.41604963	0.00000000	-0.32132411
As	2.67735125	1.57341061	-3.32916148
As	3.73089395	1.59624308	2.65448208
V	-0.00000000	-4.59002779	0.13560102
As	-1.64440388	-4.08340158	3.08229447
V	-0.00000000	-3.70064783	-2.72403946
O	2.69161959	-1.48487456	-1.41472021
O	2.92649650	-1.41785111	1.01177751
O	5.03308490	0.00000000	-0.45709594
O	1.72809201	-0.00000000	-3.53057017
O	3.18813738	0.00000000	3.38169537
O	1.46762908	-3.83828409	1.27927716
O	1.23910945	-3.89472589	-1.17713609
O	-0.00000000	-6.22596304	0.20805696
O	-0.00000000	-3.49004118	3.63981276
O	0.00000000	5.15697078	-3.46600416
V	2.62202146	-3.01846721	-0.16995966
As	2.67735125	-1.57341061	-3.32916148
As	3.73089395	-1.59624308	2.65448208
As	1.64440388	-4.08340158	3.08229447
O	-0.00000000	-5.15697078	-3.46600416
O	4.01191491	-3.86180112	-0.26246849

[$V_8As_{16}O_{40}$]
64

V	1.57794599	-3.80949862	0.00000000
O	0.00000000	-3.74890923	-1.22890876
O	2.65087914	-2.65087914	1.22890876
O	0.00000000	-3.74890923	1.22890876
O	2.65087914	-2.65087914	-1.22890876
O	2.19508796	-5.29941112	0.00000000
V	-1.57794599	-3.80949862	0.00000000
As	0.00000000	-4.23702509	-3.02149281
V	3.80949862	-1.57794599	0.00000000
As	2.99602917	-2.99602917	3.02149281
As	-0.00000000	-4.23702509	3.02149281
As	2.99602917	-2.99602917	-3.02149281
O	-2.65087914	-2.65087914	-1.22890876
O	-2.65087914	-2.65087914	1.22890876
O	-2.19508796	-5.29941112	0.00000000
O	-1.27535626	-3.07898238	-3.60355928
O	1.27535626	-3.07898238	-3.60355928
O	3.74890923	0.00000000	1.22890876
O	3.74890923	0.00000000	-1.22890876
O	5.29941112	-2.19508796	0.00000000
O	3.07898238	-1.27535626	3.60355928

O	1.27535626	-3.07898238	3.60355928
O	-1.27535626	-3.07898238	3.60355928
O	3.07898238	-1.27535626	-3.60355928
V	-3.80949862	-1.57794599	-0.00000000
As	-2.99602917	-2.99602917	-3.02149281
As	-2.99602917	-2.99602917	3.02149281
V	3.80949862	1.57794599	0.00000000
As	4.23702509	-0.00000000	3.02149281
As	4.23702509	-0.00000000	-3.02149281
O	-3.74890923	-0.00000000	-1.22890876
O	-3.74890923	-0.00000000	1.22890876
O	-5.29941112	-2.19508796	0.00000000
O	-3.07898238	-1.27535626	-3.60355928
O	-3.07898238	-1.27535626	3.60355928
O	2.65087914	2.65087914	1.22890876
O	2.65087914	2.65087914	-1.22890876
O	5.29941112	2.19508796	0.00000000
O	3.07898238	1.27535626	3.60355928
O	3.07898238	1.27535626	-3.60355928
V	-3.80949862	1.57794599	0.00000000
As	-4.23702509	0.00000000	-3.02149281
As	-4.23702509	0.00000000	3.02149281
V	1.57794599	3.80949862	0.00000000
As	2.99602917	2.99602917	3.02149281
As	2.99602917	2.99602917	-3.02149281
O	-2.65087914	2.65087914	-1.22890876
O	-2.65087914	2.65087914	1.22890876
O	-5.29941112	2.19508796	0.00000000
O	-3.07898238	1.27535626	-3.60355928
O	-3.07898238	1.27535626	3.60355928
O	-0.00000000	3.74890923	1.22890876
O	-0.00000000	3.74890923	-1.22890876
O	2.19508796	5.29941112	0.00000000
O	1.27535626	3.07898238	3.60355928
O	1.27535626	3.07898238	-3.60355928
V	-1.57794599	3.80949862	0.00000000
As	-2.99602917	2.99602917	-3.02149281
As	-2.99602917	2.99602917	3.02149281
As	0.00000000	4.23702509	3.02149281
As	0.00000000	4.23702509	-3.02149281
O	-2.19508796	5.29941112	0.00000000
O	-1.27535626	3.07898238	-3.60355928
O	-1.27535626	3.07898238	3.60355928

References:

1 M. Rasmussen, C. Näther and W. Bensch, *Zeitschrift für Anorg. und Allg. Chemie*, 2021, **647**, 992–997.

2 A. Wutkowski, N. Evers and W. Bensch, *Zeitschrift für Anorg. und Allg. Chemie*, 2011, **637**, 2205–2210.

3 R. Basler, G. Chaboussant, A. Sieber, H. Andres, M. Murrie, P. Kögerler, H. Bögge, D. C. Crans, E. Krickemeyer, S. Janssen, H. Mutka, A. Müller and H.-U. Güdel, *Inorg. Chem.*, 2002, **41**, 5675–5685.

4 A. Müller, J. Döring and H. Bögge, *J. Chem. Soc. Chem. Commun.*, 1991, 273–274.
